# Green-synthesized silver nanoparticles from *Zingiber officinale* extract: antioxidant potential, biocompatibility, anti-LOX properties, and in silico analysis

**DOI:** 10.1186/s12906-024-04381-w

**Published:** 2024-02-13

**Authors:** Tassanee Ongtanasup, Patipat Kamdenlek, Chawan Manaspon, Komgrit Eawsakul

**Affiliations:** 1 Department of Applied Thai Traditional Medicine, School of Medicine, Walailak University, Nakhon Si Thammarat, 80160 Thailand; 2 Biomedical Engineering Institute, Chiang Mai University, Chiang Mai, 50200 Thailand; 3 Biomedical Engineering and Innovation Research Center, Chiang Mai University, Chiang Mai, 50200 Thailand; 4 Research Excellence Center for Innovation and Health Products (RECIHP), Walailak University, Nakhon Si Thammarat, 80160 Thailand

**Keywords:** *Zingiber officinale*, Silver nanoparticles, Antioxidant, Biocompatibility, Anti-LOX

## Abstract

**Introduction:**

*Zingiber officinale* extract has emerged as a compelling candidate for green synthesis of nanoparticles, offering diverse applications across medicine, cosmetics, and nutrition. This study delves into the investigation of in vitro toxicity and explores the biomedical utility of green-synthesized silver nanoparticles derived from ginger extract (GE-AgNPs).

**Methods:**

We employed established protocols to evaluate in vitro aspects such as antioxidant capacity, anti-inflammatory potential, and biocompatibility of GE-AgNPs. Additionally, molecular docking was employed to assess their anti-lipoxygenase (anti-LOX) activity.

**Results:**

Our findings highlight that the extraction of ginger extract at a pH of 6, utilizing a cosolvent blend of ethanol and ethyl acetate in a 1:1 ratio, yields heightened antioxidant capacity attributed to its rich phenolic and flavonoid content. In the context of silver nanoparticle synthesis, pH 6 extraction yields the highest quantity of nanoparticles, characterized by an average size of 32.64 ± 1.65 nm. Of particular significance, GE-AgNPs (at pH 6) demonstrated remarkable efficacy in scavenging free radicals, as evidenced by an IC_50_ value of 6.83 ± 0.47 µg/mL. The results from the anti-LOX experiment indicate that GE-AgNPs, at a concentration of 10 µg/mL, can inhibit LOX activity by 25%, outperforming ginger extract which inhibits LOX by 17–18%. Notably, clionasterol exhibited higher binding energy and enhanced stability (-8.9 kcal/mol) compared to nordihydroguaiaretic acid. Furthermore, a cell viability study confirmed the safety of GE-AgNPs at a concentration of 17.52 ± 7.00 µg/mL against the L929 cell line.

**Conclusion:**

These comprehensive findings underscore the significant biomedical advantages of GE-AgNPs and emphasize their potential incorporation into cosmetic products at a maximum concentration of 10 µg/mL.

**Supplementary Information:**

The online version contains supplementary material available at 10.1186/s12906-024-04381-w.

## Introduction

The field of green nanotechnology encompasses biological processes that enable the synthesis, manipulation, and utilization of materials within the 1–100 nm range, offering cost-effective and straightforward methodologies [1]. The term "green nanotechnology" refers to the utilization of natural resources to mitigate adverse environmental impacts, safeguard human health, and reduce the financial burden associated with nanoparticle production. Within this field, considerable research efforts have been focused on the development of nanomaterials derived from plant sources. Plant extracts contain a diverse array of phytochemicals, including alkaloids, terpenes, saponins, phenols, alcohols, and proteins, which serve dual roles as both reducing agents and capping agents. Isolation of these phytochemicals enables the enhancement of nanomaterials' size and morphology, thereby facilitating their application in pharmacological and biological research endeavors [2, 3]. Anti-inflammatory drugs play a critical role in the treatment of pain. Consequently, the practicality of employing anti-inflammatory medications to relieve the pain, necessitates the exploration and development of innovative therapeutic agents [4, 5]. Given the challenges associated with traditional painkillers, such as diminished efficacy leading to the use of high doses, considerable scientific efforts have been devoted to the design and enhancement of novel anti-inflammatory compounds with unique mechanisms of action for clinical applications [6]. Pharmaceutical companies have undertaken efforts to modify existing anti-inflammatory drugs and have identified a few promising anti-inflammatory compounds as potential replacements for conventional treatments [7]. The remarkable anti-inflammatory properties of metallic nanoparticles (NPs) can be attributed to their large surface area-to-volume ratio. Among these nanoparticles, silver nanoparticles (AgNPs) have demonstrated exceptional efficacy as anti-inflammatory agents, primarily due to their enhanced contact with target protein resulting from their expansive surface area. AgNPs effectively bind to and penetrate through skin, where they interact with LOX enzymes and compounds. This interaction disrupts LOX enzyme, ultimately leading to reduce pain. Additionally, the release of cationic ions by AgNPs provides antioxidant activity [8].

Laser ablation [9], gamma irradiation [10], and the employment of chemical agents [11] as reducing and capping agents represent only a subset of the diverse methodologies available for the synthesis of AgNPs. The exorbitant costs associated with these approaches, coupled with the use of hazardous chemical agents, present significant impediments due to their adverse impacts on human health and the environment [12]. Within the field of nanotechnology, the emergence of green synthesis has garnered considerable attention as a viable alternative for NP fabrication. Green synthesis NPs have demonstrated remarkable efficacy against primary biofilms. Notably, the utilization of plants as a source for NP synthesis offers distinct advantages over conventional physical and chemical methods [13]. In recent years, the utilization of plant extracts for NP synthesis [14, 15] has gained considerable traction owing to their widespread availability, environmentally sustainable characteristics, ease of implementation, and the diverse array of secondary metabolites they harbor, which can be harnessed as potent reducing agents [16].

Ginger (*Zingiber officinale* Roscoe), a perennial herbaceous plant of the Zingiberaceae family, is distinguished by its robust tuberous rhizomes. *Zingiber officinale* Roscoe, commonly referred to as ginger, is a perennial plant characterized by its climbing growth pattern. Ginger extract is replete with a diverse array of natural compounds, each possessing distinct physiological effects. The specific chemical composition of ginger is contingent upon its geographical origin and the state of the rhizomes, whether they are fresh or dried. Ginger extracts have been identified to contain an extensive spectrum of more than 400 distinct chemical constituents [17], with ongoing discoveries of additional compounds. However, it is noteworthy that only a limited subset of these chemical constituents has been subjected to rigorous investigation concerning their potential pharmacological properties. Among the recognized chemical constituents present in ginger are lipids (fats), terpenes (aromatic compounds), carbohydrates (sugars), and phenolic compounds (naturally occurring plant compounds). From a chemical perspective, these constituents may be classified into two primary categories: those contributing to the flavor profile of ginger and those responsible for its pungent attributes. Numerous pungent compounds within ginger, including but not limited to gingerols, shogaols, zingerones, gingerdiols, gingerdione, and paradols, are well-documented for their diverse range of pharmacological effects [18], and these compounds have been the subject of extensive scientific inquiry. An emerging trend among individuals afflicted with chronic inflammatory conditions is the pursuit of relief from symptoms and the adoption of natural remedies for preventative purposes [19]. The orchestration of the inflammatory response, encompassing the initiation of inflammation, the recruitment and activation of immune cells, and the subsequent resolution of the inflammatory process, is intricately regulated through a complex interplay involving inflammatory cells and a diverse array of chemical mediators. Numerous scientific investigations have substantiated the effectiveness of various chemical compounds present in ginger in ameliorating symptoms associated with chronic inflammatory ailments. These bioactive compounds exert their therapeutic actions primarily by inhibiting the production of prostaglandins via the cyclooxygenase (COX) and lipoxygenase (LOX) pathways [20]. The historical utilization of ginger infusions for the management of conditions such as rheumatism and arthritis has prompted extensive research into the anti-inflammatory mechanisms of the plant's secondary metabolites. According to several researchers [21, 22], the anti-inflammatory properties of 6-gingerol can be attributed to its capacity to diminish pro-inflammatory cytokine levels and impede antigen presentation by macrophages stimulated by lipopolysaccharides (LPS). Liang et al. [23] conducted a study demonstrating that shogaols and all gingerols exhibit a dose-dependent reduction in the production of nitric oxide (NO) in RAW 264.7 cells treated with LPS. Thus, the integration of ginger extract as a reducing agent in the synthesis of AgNPs serves a dual purpose of enhancing anti-inflammatory action and minimizing the overall material requirements for anti-inflammatory product development. Notwithstanding the feasibility of utilizing ginger for AgNPs synthesis, investigations exploring ginger extracts with elevated concentrations of phenolic and flavonoid constituents are currently lacking. This research gap arises from the recognition that the extraction of biologically active chemical constituents from the phenolic and flavonoid classes is of paramount importance in attaining exceptional properties as reducing agents [24]. Furthermore, the active compounds present in ginger, which exhibit significant phenolic and flavonoid content, hold promise for enhancing the inhibition of inflammation [25]. The utilization of computer-based studies can play a pivotal role in reducing errors inherent in experimental procedures and increasing the likelihood of successful outcomes, thereby circumventing the need for extensive trial and error. Consequently, an investigation into the efficacy of ginger's main compounds in inhibiting inflammatory production, achieved through the use of molecular docking processes to impede the activity of the LOX enzyme, contributes to a deeper understanding of the effectiveness of ginger.

The primary objective of this research is to synthesize AgNPs through the implementation of environmentally friendly extraction methods involving variations in pH using ethyl acetate and ethanol, along with chemical synthesis techniques. The utilization of ginger root extract was applied as the reducing agent in the aforementioned procedure. Prior to utilizing ginger extract as a reducing agent, it is crucial to conduct a thorough assessment of the phenolic and flavonoid composition, as well as to ascertain the chemical constituents within the ginger extract through the application of Gas Chromatography-Mass Spectrometry/Mass Spectrometry (GC–MS/MS) analysis. Conducting a thorough examination is vital to acquire an intricate comprehension of the composition and concentration of the bioactive elements included in the ginger extract. The examination of the characteristics of the investigated compounds in relation to their capacity to hinder the LOX enzyme is of utmost importance. The utilization of sophisticated computational methodologies, such as molecular docking, can facilitate the attainment of this objective. This methodology provides a comprehensive analysis of the interaction between the chemicals present in ginger extract and the LOX enzyme, thereby offering valuable insights into their possible inhibitory effects on the formation of inflammation. Through the implementation of meticulous investigations, a thorough comprehension of the chemical makeup of ginger extract and its capacity as an inhibitor of inflammatory production can be attained. The acquisition of this knowledge enhances scientific comprehension within the discipline and enables the formulation of efficient approaches for harnessing ginger extract as a reducing agent, displaying potential applications across several fields. In addition, it is crucial to evaluate the antioxidant potential of the ginger extract in relation to the scavenging of 2,2-diphenyl-1-picrylhydrazyl (DPPH) and 2,2'-azino-bis(3-ethylbenzothiazoline-6-sulfonic acid) (ABTS) radicals. The synthesized AgNPs were subjected to meticulous characterization using dynamic light scattering (DLS), transmission electron microscopy (TEM), ultraviolet–visible spectroscopy (UV–Vis), and Fourier-transform infrared spectroscopy (FTIR). Additionally, comprehensive biocompatibility assessments encompassing cytotoxicity were performed using L929 cells to ensure the safety and viability of the AgNPs.

## Materials and methods

### Materials

In this research, materials were divided into three groups as follows: Group 1, which involved experiments related to chemical reactions, used the following equipment: silver nitrate (AgNO_3_), 2,2-diphenyl-1-picrylhydrazyl (DPPH), 2,2′-azino-bis(3-ethylbenzothiazoline-6-sulfonic acid) diammonium salt (ABTS), potassium persulfate (K_2_S_2_O_8_), Folin-Ciocalteu reagent, aluminium chloride (AlCl_3_), sodium carbonate (Na_2_CO_3_), quercetin, gallic acid, and Trolox. These materials were purchased from Sigma-Aldrich (Missouri, USA). The rhizomes of *Zingiber officinale* were procured from Charoensuk Osod, an authorized traditional medicine dispensary situated in Nakhon Pathom, Thailand.

Group 2, which involved studies with cell lines and enzymes, used the following equipment: Dimethyl sulfoxide (DMSO), 3-(4,5-dimethylthiazol-2-yl)-2,5-diphenyl-2H-tetrazolium bromide (MTT), and comprehensive cell culture materials were utilized to support these studies, in accordance with established laboratory protocols. These materials were purchased from Merck (Darmstadt, Germany). Lipoxygenase (LOX) enzyme and linoleic acid were obtained from Sigma-Aldrich (Missouri, USA).

Group 3, which involved studies and analysis through computer systems, utilized the following software programs: AutoDock 1.5.6, Python 3.8.2, MGLTools 1.5.4, Discovery Studio-2017, ArgusLab 4.0.1, and Avogadro. These software programs were used under the control of a computer system with the following specifications with processor: Intel Xeon-E5-2678v3 12C/24 T CPU @ 2.50 GHz—3.10 GHz, system memory: 32 GB RAM DDR4-2133 RECC, graphics processing: VGA GTX 1070 TI 8G, system type: 64-bit operating system, and operating System: Windows 10.

### Collection of plant

The rhizomes of *Zingiber officinale* were procured from Charoensuk Osod, an authorized traditional medicine dispensary situated in Nakhon Pathom, Thailand. To ensure the authenticity of the plant material, a voucher specimen with the reference number TTM-c No. 1000704 was obtained and subsequently deposited at the Thai Traditional Medicine Herbarium, which is under the jurisdiction of the Department of Thai Traditional and Alternative Medicine in Bangkok, Thailand. The acquired dried rhizomes were finely powdered using a grinder. Subsequently, the resulting powder was carefully stored in a glass bottle at room temperature until it was ready for further utilization.

### Plant extraction

#### Water extraction

To perform the water extraction following previous research [26], a mixture of herbal powder weighing 400 g was combined with one liter of warm deionized water. The resulting herb solution was subjected to heating on a hot plate, maintaining a temperature of 100 °C for a duration of 15 min. To compensate for the water absorption by the herb, an additional 1000 mL of hot water was introduced into the solution. Subsequently, the solution was boiled down until its volume reduced to approximately one-third of the initial amount. To eliminate any solid particles, the solution underwent filtration using Whatman No. 1 filter paper, after which it was stored at a temperature of -20°C. Freeze-drying of the frozen samples was conducted using an Eyela FDU-2100 freeze-dryer (Bohemia, New York, USA.)

#### The extraction of *Z. officinale* rhizomes by ethanol and ethyl acetate

Initially, 400 g of *Z. officinale* rhizomes underwent individual extraction using a 50:50 mixture of ethanol and ethyl acetate. The pH of the extraction solution was adjusted to fall within the range of 5.0 to 7.0 by adding 1.0 M NaOH or HCl as needed. The extraction process employed the maceration technique and lasted for a period of three days. Subsequently, the resulting liquid was meticulously filtered through Whatman No. 1 filter paper, and the collected filtrate was then subjected to evaporation using a rotary evaporator (Heidolph Basic Hei-VAP ML, Schwabach, Germany), resulting in the formation of a highly viscous extract dissolved in cosolvent. To ensure optimal extraction efficiency, the maceration process was repeated two more times following the same protocol. The residual ethanol present in the herbal material was eliminated by subjecting it to further evaporation in a vacuum drying chamber (Binder VD 23, Tuttlingen, Germany). Evaporation continued until a consistent weight was attained, indicating the complete removal of all remaining cosolvent.

### Preparation of ginger extract for yield analysis

The percentage yield of the aqueous extract was calculated according to Eq. (1). These procedures were repeated for powdered rhizomes of *Z. officinale* that were extracted using various aqueous and cosolvent solutions at different pH levels.

1 $$\mathrm{Percentage}\;\mathrm{yield}=\frac{\text{W}2-\text{W}1}{\text{W}0}\times100$$

"W0" represents the weight of the dried ginger sample, "W1" indicates the weight of the container, and "W2" corresponds to the combined weight of the dried ginger extract and the container.

### Quantity of phenolic compounds

The determination of total phenolic content was conducted using the Folin-Ciocalteu method, which was referenced in a previous study [27]. In brief, a concentration of 1 mg/mL for the ginger extracts was achieved by diluting a stock concentration with either distilled water or methanol. Subsequently, a mixture comprising 100 µL of 0.1 M Na_2_CO_3_ solution and 100 µL of Folin-Ciocalteu reagent at a concentration of 10% was added to individual wells of a 96-well plate. The plate was then incubated at room temperature for one hour. Following the incubation period, the absorbance of the resulting mixture was measured at 750 nm. A series of gallic acid solutions with concentrations ranging from 6.25 to 800 µg/mL were employed to generate a standard curve. The total phenolic content was quantified in terms of gallic acid equivalents (GAE) expressed in milligrams per gram of the dried plant extract.

### Quantity of flavonoid compounds

The quantification of total flavonoid concentration in the ginger extract was conducted employing a well-established protocol [28]. Concisely, the GA extract (at a concentration of 1 mg/mL) or quercetin standard solutions (ranging from 6.25 to 200 µg/mL) were combined with 100 µL of a 2% AlCl_3_ solution in methanol and incubated at room temperature for 30 min. Subsequently, the absorbance of the resulting solution was measured at 415 nm against a blank. The obtained values were expressed as quercetin equivalents (QE) per gram of dried plant extract, serving as a measure of total flavonoid content.

### 2,2-Diphennyl-1-picrylhydrazyl (DPPH) radical scavenging activity

In accordance with a standardized methodology [29, 30], the ginger extract was assessed for its capacity to scavenge free radicals. briefly, 20 µL of the ginger extract or Trolox solution in ethanol was combined with 180 µL of freshly prepared DPPH solution. The resulting mixture was vigorously shaken and allowed to incubate at room temperature, shielded from direct sunlight. After a 30 min incubation period, the absorbance of the solution was measured at 517 nm. This experimental procedure was performed in triplicate to ensure reliability. The quantification of results involved expressing the data as Trolox equivalents (TE) per gram of dry plant extract. The DPPH-scavenging activity was determined through the utilization of Eq. (2), enabling the calculation of the observed outcomes.

2 $$\%\;\mathrm S\mathrm c\mathrm a\mathrm v\mathrm e\mathrm n\mathrm g\mathrm i\mathrm n\mathrm g\;\mathrm a\mathrm c\mathrm t\mathrm i\mathrm v\mathrm i\mathrm t\mathrm y=\frac{\mathrm{Abs}\;\mathrm{of}\;\mathrm{control}-\left(\mathrm{Abs}\;\mathrm{of}\;\mathrm{sample}-\mathrm{Abs}\;\mathrm{of}\;\mathrm{blank}\right)}{\mathrm{Abs}\;\mathrm{of}\;\mathrm{control}}\times100$$

The graph plot depicting the free radical scavenging activity was utilized to determine the IC_50_ value, denoting the concentration required to inhibit 50% of the DPPH activity.

### ABTS^.+^ radical scavenging activity

The assessment of the extract's ability to scavenge free radicals was conducted utilizing the ABTS decolorization test [31]. Following the mixture's incubation in darkness for 12–16 h at room temperature, the ABTS radical cation (ABTS^•+^) was prepared by adding 2.45 mM potassium persulfate to a solution of 7 mM ABTS. To achieve a final absorbance of 0.70 ± 0.02, methanol was employed to dilute the ABTS reagent. A combination of 20 µL of extract or Trolox and 180 µL of the ABTS reagent was tested for 45 min incubation. The experiments were conducted on a minimum of three separate occasions. Utilizing Eq. (3), the extent of absorbance reduction at 734 nm was calculated as a percentage to determine the level of inhibition.

3 $$\%\mathrm S\mathrm c\mathrm a\mathrm v\mathrm e\mathrm n\mathrm g\mathrm i\mathrm n\mathrm g\;\mathrm a\mathrm c\mathrm t\mathrm i\mathrm v\mathrm i\mathrm t\mathrm y=\frac{\mathrm{Abs}\;\mathrm{of}\;\mathrm{control}-\left(\mathrm{Abs}\;\mathrm{of}\;\mathrm{sample}-\mathrm{Abs}\;\mathrm{of}\;\mathrm{blank}\right)}{\mathrm{Abs}\;\mathrm{of}\;\mathrm{control}}\times100$$

The IC_50_, representing the concentration at which 50% inhibition of ABTS^.+^ activity occurs, was determined through the construction and analysis of a concentration–response curve for ABTS^.+^.

### GC–MS/MS analysis

Gas chromatography-mass spectrometry (GC–MS/MS) was employed as the analytical technique of choice to investigate the volatile and semi-volatile constituents inherent in ginger. The analysis was meticulously conducted utilizing an Agilent HP-5MS column (5% phenyl methyl Silox, 30 µm × 250 µm × 0.25 µm) in conjunction with helium as the carrier gas. The analytical instrument employed for this investigation was the GC/MS GC 7890 B, MSD 5977B, which was manufactured by Agilent Technologies, United States. The analysis was performed under meticulously controlled experimental settings, wherein certain parameters were set, including a flow rate of 1 mL/min, a pressure of 7.6522 psi, an average velocity of 36.445 cm/s, a holdup duration of 1.3719 min, and a post-run flow rate of 1 mL/min. The sample was introduced into the system using a split mode, with an injection volume of 1 µL. The oven's temperature profile adhered to a carefully planned 69 min cycle, beginning at an initial temperature of 50 °C. This temperature was sustained for a period of 5 min to establish thermal equilibrium. Following this, the temperature was gradually increased in a controlled manner at a rate of 5 °C per min until it attained a final temperature of 320 °C. At this final temperature, the system was held steady for a period of 10 min to allow for optimal separation and detection of the analytes of interest. To identify the chemicals present in ginger, the data obtained from the analysis was compared against the chemical libraries available in the Agilent MassHunter Quantitative Analysis software (version B.09.00). A match with a score of 90% or higher was considered acceptable for identifying the compounds [32].

### The use of *Zingiber officinale* aqueous and cosolvent extracts in the production of silver nanoparticles

The experimental procedure began with the preparation of 2 mM aqueous silver nitrate solutions by dissolving 6.5 mg of silver nitrate in 19 mL of deionized water as described in the petty patent number 2303001991. The formation of silver nanoparticles (AgNPs) was evaluated through spectrophotometric measurements based on color change, serving as an indicative parameter. The synthesis of AgNPs followed established protocols with slight modifications as referenced. Subsequently, meticulous preparation of aqueous and organic ginger extracts with pH values of 5, 6, and 7 was maintained for synthesis. Each extract, comprising 1 mL, was gradually added to the previously prepared 2 mM silver nitrate solution (19 mL) under ambient conditions, employing continuous stirring for a duration of 30 min. The resulting mixtures in the beaker were carefully adjusted to a pH of 8 using a pH meter and sodium hydroxide (0.1 M) as the requisite reagent. Noteworthy, the silver nitrate solutions were subjected to a 30 min sonication process in a light-protected environment to prevent any unintended photoactivation. The reduction of silver ions to AgNPs was initially identified by a discernible change in color, characterized by a shift to a yellowish or dark brownish shade.

### Characterization

The characterization of the as-prepared silver nanoparticles (AgNPs) involved various analytical techniques [33]. UV–visible spectroscopy was performed using a Nanodrop 2000 spectrophotometer to study the absorbance properties of AgNPs in the wavelength range of 200–800 nm [34]. Dynamic light scattering (DLS) measurements were conducted at 25 ± 0.1 °C using a Zetasizer Nano ZS instrument (Malvern, UK) to determine the size and zeta potential of the AgNPs. In order to conduct a more comprehensive analysis of the dimensions and structure of the AgNPs, the utilization of transmission electron microscopy (TEM) was employed. The transmission electron microscopy (TEM) study was conducted utilizing a JEM-2010 microscope, which was operated at an acceleration voltage of 200 kilovolts (kV). This methodology facilitated the verification and comprehensive analysis of the dimensions and morphology of the silver nanoparticles (AgNPs). In order to confirm the existence of silver nanoparticles and evaluate their elemental composition, an Energy Dispersive X-ray (EDX) analysis was conducted. This methodology offers insights on the elemental makeup of the nanoparticles. Furthermore, an investigation using Fourier transform infrared spectroscopy (FTIR) was performed on the freeze-dried powder derived from the AgNP solution. The analysis was conducted utilizing a Bruker Tensor 27 apparatus, utilising KBr pellets as the sample medium, and including a wavelength range spanning from 400 to 4000 cm^-1^. The utilization of FTIR analysis facilitated the comprehension of the chemical properties and functional groups that are present inside the AgNPs. The combination of these analytical techniques for the characterization of the AgNPs was achieved.

### Cell culture of L929 fibroblasts

Murine L929 fibroblast cells were obtained from Japanese Collection of Research Bioresources Cell Bank (JCRB) and cultured in Dulbecco's Modified Eagle Medium (Gibco, USA) supplemented with 10% Fetal Bovine Serum (FBS, Gibco, USA) and 1,000 U/mL of penicillin & 100 µg/mL of streptomycin (Gibco, USA) at a controlled temperature of 37°C with 5% CO_2_ atmosphere. Upon reaching approximately 80% confluence, the cells were subcultured using Trypsin–EDTA solution and subsequently utilized in the experimental procedures.

### In vitro cytotoxicity

Chiang Mai University's Institutional Biosafety Committee (IBC) granted authorization for the utilization of an L929 cell line (clearing number: CMUIBC01-66/01) prior to initiating the research. The cytotoxicity of GE-AgNPs against L929 fibroblast cells was assessed employing the MTT assay [35–37]. L929 fibroblast cells were seeded onto a 24-h cell culture plate and incubated under standard conditions at 37°C with 5% CO_2_ for a period of 24 h. Subsequently, the cells were treated with varying concentrations of GE-AgNPs (6.25, 12.5, 25, 50, 100, and 1,000 µg/mL). Simultaneously, the ginger extract was prepared at concentrations ranging from (6.25, 12.5, 25, 50, 100, and 200 µg/mL), along with the corresponding control (untreated), and the cells were incubated for an additional 24 h. Following rinsing of the cells with phosphate-buffered saline (PBS), they were exposed to MTT solution (0.5 mg/mL) for 3 h. The resulting insoluble formazan crystals were subsequently dissolved in 100 µL of dimethyl sulfoxide (DMSO) solution. The absorbance of each sample was analyzed spectrophotometrically at 570 nm using a microplate reader (TECAN, Infinite 200 Pro M Plex, Switzerland).

### Molecular docking to evaluate the inhibition of LOX

The molecular docking employed was carried out following the procedures outlined in the previous research [38–41], summarized as follows: Avogadro, a sophisticated 3D visualization and modeling software, was crucial for designing and optimizing product structures in this study. The crystal structures of lipoxygenase (LOX) were obtained from the respected Protein Data Bank (PDB: 7LAF; https://www.rcsb.org/). The protein structures were prepared by removing inhibitor ligands and water, followed by adding polar hydrogen atoms for completeness. Molecular docking analysis was performed to evaluate LOX inhibition. Target enzymes were placed in grid boxes with a 1 Å interval to optimize binding site interactions. The grid box dimensions were 120 × 58x126, centered at (x, y, z) = (-46.211, 9.567, 530.685), encompassing the coverage of all pocket binding positions. The molecular docking analysis utilized advanced software, including AutoDock [42], Autodock Vina [43] and Arguslab [44], known for accurate ligand–protein interaction predictions. Visualization tools like Biovia Discovery Studio Visualizer were employed to gain insight into ligand-receptor interactions and identify potential LOX inhibitors.

### In-vitro lipoxygenase inhibitory activity

The samples were evaluated for their lipoxygenase (LOX) inhibitory potential using LOX as the enzyme and linoleic acid as the substrate. The samples were diluted in DMSO to a final concentration of 10 and 100 µg/mL (25 µl) and mixed with 400 U/mL (975 µl) of LOX enzyme in 0.2 M borate buffer (pH 9.0) [45, 46]. The reaction was initiated by adding 1 mL of linoleic acid (250 µM) in 0.2 M borate buffer, pH 9.0. The absorbance of the reaction was then measured at 234 nm after 5 min using a microplate reader (TECAN, Infinite 200 Pro M Plex, Männedorf, Switzerland) [45]. The control was conducted using only DMSO solvent. Gallic acid (10 and 100 µg/mL) was used as the reference active compounds. The percentage of LOX inhibitory activity was calculated using the following Eq. 4.

4 $$\mathrm{LOX}\;\mathrm{inhibitory}\;\mathrm{activity}\;\left(\%\right)=\frac{\mathrm{Absorbance}\;\mathrm{of}\;\mathrm{control}-\mathrm{Absorbance}\;\mathrm{of}\;\mathrm{sample}}{\mathrm{Absorbance}\;\mathrm{of}\;\mathrm{sample}}\times100$$

### Statistical analysis

A one-way analysis of variance (ANOVA) was performed to examine the statistical significance of the data. To determine significant differences between groups, a post hoc Tukey's Honestly Significant Difference (HSD) test was applied. A significance level (α) of 0.05 was chosen, indicating that differences with a *p-value* below this threshold were considered statistically significant. All data are presented as mean ± standard deviation (SD) and were analyzed using SPSS version 20.0 statistical software.

## Results and discussion

### The analysis of ginger extract

Table 1 illustrates a notable disparity in the extraction yields of ginger rhizome extracts between hot water extraction and ethanol: ethyl acetate extraction at various pH levels. The use of hot water consistently resulted in significantly higher extraction yields of ginger. The total concentration of phenolic compounds exhibited substantial variations among the ginger extracts obtained through different extraction techniques. Specifically, the ginger extract obtained by ethanol extraction at pH 6 displayed the highest levels of total phenolics (176.65 ± 11.53 mg GAE/g extract) and total flavonoids (76.10 ± 1.98 mg QE/g extract) compared to the other extraction conditions (Table 2). Notably, the ginger extract with elevated concentrations of total flavonoids corresponded to a greater abundance of total phenolic components. Conversely, the total phenolic and flavonoid content in the ginger extract experienced a decline during hot water extraction (Table 2). This observation suggests that the hot water extraction method may contribute to the degradation of phenolic compounds in ginger. Consistent with this, Singh and Saldaa [47] have found that elevated temperatures significantly reduce the extraction of phenolic chemicals from plant materials. Thus, for the optimal extraction of phenolics and flavonoids from the ginger extract, it is recommended to employ ethanol: ethyl acetate extraction at a pH of 6. The several research studies [48, 49] corroborate the notion that ethanol or ethyl acetate can effectively be utilized for the extraction of bioactive compounds, including phenolics and flavonoids.

**Table 1:** The yield percentage of *Zingiber officanale* extracts from water and mixing solvents between 50:50 v/v ethanol and Ethyl acetate in different pH

Extraction	Yield
Water	11.83 ± 0.49%
pH 5 (ethanol + Ethyl acetate)	9.55 ± 0.16%
pH 6 (ethanol + Ethyl acetate)	8.49 ± 0.14%
pH 7 (ethanol + Ethyl acetate)	7.89 ± 0.13%

**Table 2:** The quantity of phenolic and flavonoid compounds of ginger extract by water and mixing solvents between 50:50 v/v ethanol and Ethyl acetate in different pH

Parameters	Amount			
	**Water**	**pH 5**	**pH 6**	**pH 7**
Total phenolic (mg GAE/g)	50.87 ± 6.78	163.85 ± 11.61	176.65 ± 11.53	154.03 ± 14.32
Total flavonoid (mg QE/g)	9.76 ± 0.74	68.31 ± 1.54	76.10 ± 1.98	68.12 ± 1.99
DPPH (mg TE/g)	32.33 ± 7.92	120.69 ± 1.64	132.86 ± 0.46	117.47 ± 0.92
DPPH (%/mg)	23.21 ± 4.67	75.35 ± 0.97	82.53 ± 0.28	73.45 ± 0.55
IC_50_ of DPPH (µg/mL)	1,592.5 ± 38.23	195.67 ± 3.06	83.20 ± 0.82	121.03 ± 5.44
ABTS (mg TE/g)	14.08 ± 1.60	102.65 ± 3.87	108.05 ± 0.89	100.15 ± 3.80
ABTS (%/mg)	13.70 ± 1.14	77.04 ± 2.77	80.91 ± 0.64	75.26 ± 2.72
IC_50_ of ABTS (µg/mL)	673.43 ± 15.91	146.97 ± 1.62	70.78 ± 1.68	108.78 ± 3.60

### Antioxidant activity

**Fig. 1:**
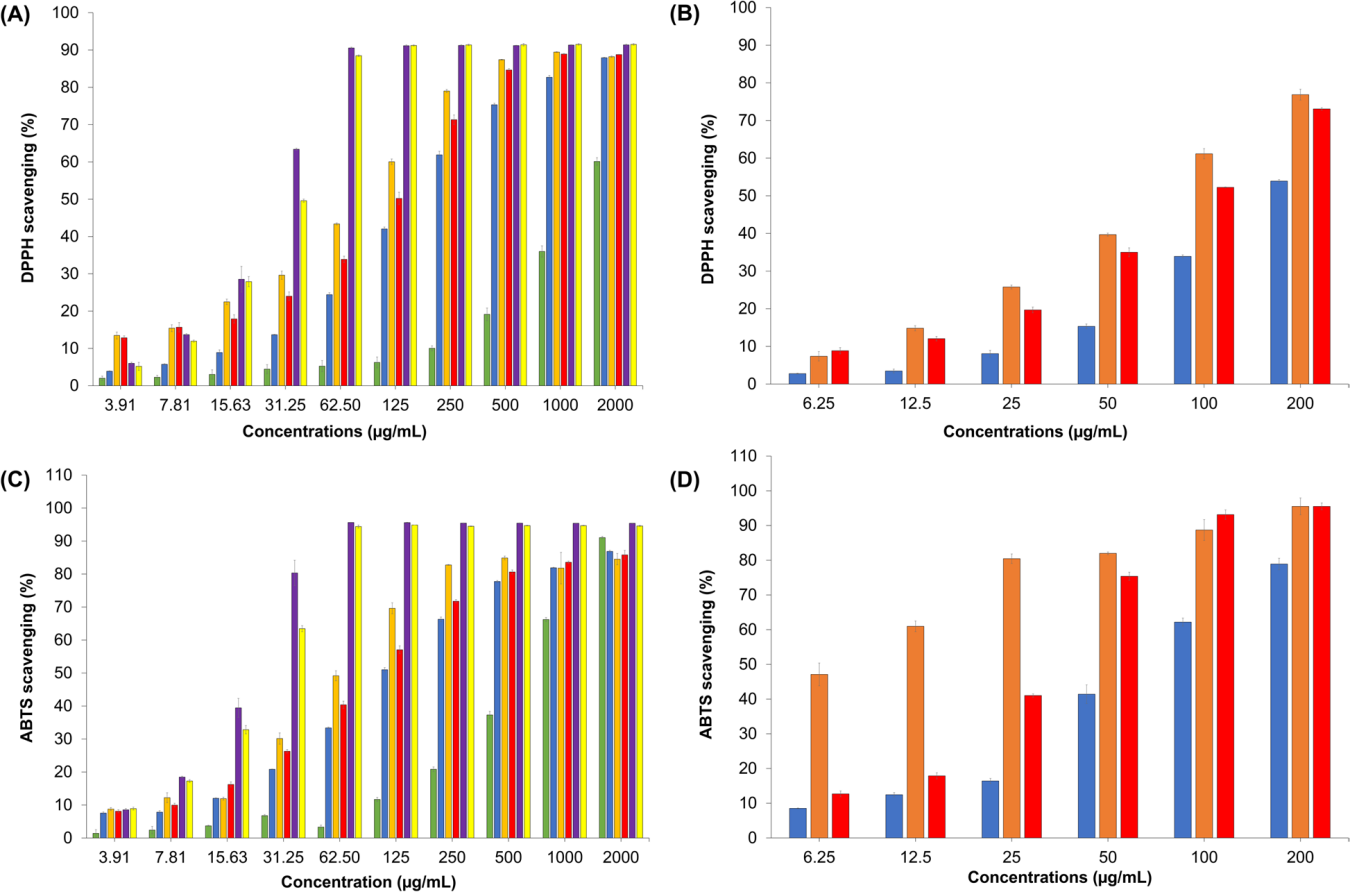
**A** DPPH radical scavenging activity of ginger extracts, **B** DPPH radical scavenging activity of GE-AgNPs, **C** ABTS radical scavenging activity of ginger extracts, **D** ABTS radical scavenging activity of GE-AgNPs. Standard deviations, calculated from triplicate determinations of each concentration, are represented by the bars. Trolox (yellow) and ascorbic acid (purple) served as references, while red, orange, blue, and green bars corresponded to pH *7*, pH *6,* pH *5* of ethyl acetate: ethanol cosolvents, and water extract, respectively

Throughout history, aromatic and medicinal botanicals have been extensively utilized for their therapeutic properties, owing primarily to the presence of bioactive compounds within them [50]. The investigation of secondary metabolites derived from these plants gained considerable prominence and became a central focus of scientific research subsequent to the advent of spectroscopy during the nineteenth century [51]. Consequently, the synthesis of nanoparticles utilizing these plant sources has garnered significant attention, offering a wide range of biological functionalities [50]. The assessment of GE-AgNPs' scavenging activity against free radicals was conducted through the utilization of the 1,1-diphenyl-2-picrylhydrazyl (DPPH) assay, as represented in Fig. 1. The results obtained from the evaluation of radical scavenging capacity employing the 2,2'-azino-bis(3-ethylbenzothiazoline-6-sulfonic acid) diammonium salt (ABTS +) assay are presented in Fig. 1. The findings presented in Table 3 demonstrate that all variations of GE-AgNPs exhibited substantially higher activity compared to the extract in the DPPH and ABTS + experiments. Nevertheless, it is imperative to observe that the extract demonstrated comparatively diminished efficacy in its entirety in contrast to Trolox. Singularly, among the studied GE-AgNPs, the one facilitating the synthesis of ginger extract under pH 6 conditions manifested superior proficiency in free radical inhibition relative to Trolox utilization. This particular GE-AgNPs (pH 6) evinced an IC_50_ value of 6.83 ± 0.47 for ABTS + , as delineated in Table 3. The results differ from the GE-AgNPs synthesized with water-based ginger extract. It was found that this resulted in an increased production of free radicals. Therefore, using ginger extract with a pH of 6 for GE-AgNPs synthesis is likely the most suitable for antioxidant purposes.

**Table 3:** Assessment of the radical scavenging potential of ginger extract was conducted using DPPH and ABTS assays, employing both aqueous and solvent mixtures consisting of ethanol and ethyl acetate in a 50:50 v/v ratio at various pH levels. The results were juxtaposed with the antioxidant standards, Ascorbic acid and Trolox

Compounds	DPPH (IC_50_) ± SD	ABTS (IC_50_) ± SD
Ascorbic	24.78 ± 0.86	18.95 ± 0.83
Trolox	29.31 ± 0.37	23.83 ± 0.06
GE (pH5)	195.67 ± 3.06	146.97 ± 1.62
GE-AgNPs (pH5)	177.20 ± 1.51	74.67 ± 2.23
GE (pH6)	83.20 ± 0.82	70.78 ± 1.68
GE-AgNPs (pH6)	74.10 ± 1.73	6.83 ± 0.47
GE (pH7)	121.03 ± 5.44	108.78 ± 3.60
GE-AgNPs (pH7)	97.70 ± 0.91	30.72 ± 0.05

When comparing GE-AgNPs, it has been observed that those synthesized through ethanol and ethyl acetate extraction exhibit superior antioxidant properties compared to those synthesized through water extraction. This discrepancy can be attributed to the presence of crucial compounds, namely *6*-shogaol, *6*-isoshogaol, *8-*shogaol, and *1-(4*-hydroxy-*3*-methoxyphenyl)tetradec-*4*-en-*3*-one, which were identified and quantified through GC–MS/MS and PASS analysis in the ethanol extract. These compounds exhibit notable efficacy in inhibiting free radicals. Notably, the ethanol and ethyl acetate extract showcases a substantially higher proportion of these compounds, reaching up to *23.66%* as shown in Table S3-S17. Conversely, the water extract of ginger contains solely *6*-shogaol, albeit at a lower proportion of *11.56%* as shown in Table S3-S17.

Based on the data presented in Table 3, it was found that ginger extract at pH 6 among all pH yielded the best results in combating free radicals, resulting in the highest antioxidant activity when used for synthesizing silver nanoparticles. The utilization of these nanoparticles holds promising potential in the fields of pharmaceuticals and food industries. It is worth noting that this study represents the first documented instance of synthesizing silver nanoparticles (AgNPs) using a pH 6 ethanol:ethyl acetate cosolvent, which incorporates ginger extract as a crucial component.

### GC–MS/MS analysis

The analysis was performed on ginger extracts obtained through the utilization of a 50% ethanol and 50% ethyl acetate mixture, as well as water. A total of 64 compounds (Table S1) were obtained from the cosolvent, while 8 compounds (Table S2) were obtained from water, all with match scores over 90%. Figures S1 and S2 present the gas chromatography-mass spectrometry (GC–MS) chromatograms illustrating the composition of the ginger rhizome extract obtained using water and cosolvent, respectively. The comprehensive analysis of the cosolvent extracts led to the isolation and identification of 237 distinct phytocompounds with known chemical structures. In comparison, the aqueous extract yielded 67compounds, also subjected to isolation and identification.

A total of fourteen chemicals were successfully extracted using a cosolvent, possessing a match score of 90 or higher and concentrations exceeding 1%. To identify these substances, mass spectrometry combined with gas chromatography (GC) was employed. The mass spectra information of these chemicals was meticulously compiled by cross-referencing them with entries in the Agilent MassHunter Quantitative Analysis spectrum database. Through this analysis, the primary compound was identified by its distinctive fragmentation pattern at a retention time of 41.3354, unequivocally characterized as 6-gingerol. Furthermore, the compound exhibiting the second highest concentration, detected at a retention time of 39.7255, was conclusively determined to be 6-shogaol. Zingiberene, a notable compound, was detected at a retention time of 23.7917. Another compound, specifically 4-(3-hydroxy-2-methoxyphenyl)butan-2-one, exhibited a retention time of 27.3058. The remaining chemicals in this comprehensive analysis encompass 1-(4-hydroxy-3-methoxyphenyl)tetradec-4-en-3-one, 8-shogaol, diacetoxy-6-gingerdiol, 6-isoshogaol, clionasterol, (S)-8-gingerol, 3-decanone, and 1-(4-hydroxy-3-methoxyphenyl).

In contrast, the aqueous extract of ginger revealed 67 components. Table S3 has been firmly established that water extraction can successfully yield 8 compounds, meeting the stringent criteria of a match score exceeding 90% and a concentration surpassing 1%. The primary constituents identified in the aqueous ginger extract were found to be 5-hydroxy-1-(4-hydroxy-3-methoxyphenyl)decan-3-one as known as 6-gingerol (27.69%), exemplifying their prominence and significance in the composition of the extract.

### Silver nanoparticle characterization

#### UV–visible (vis) spectroscopy

**Fig. 2:**
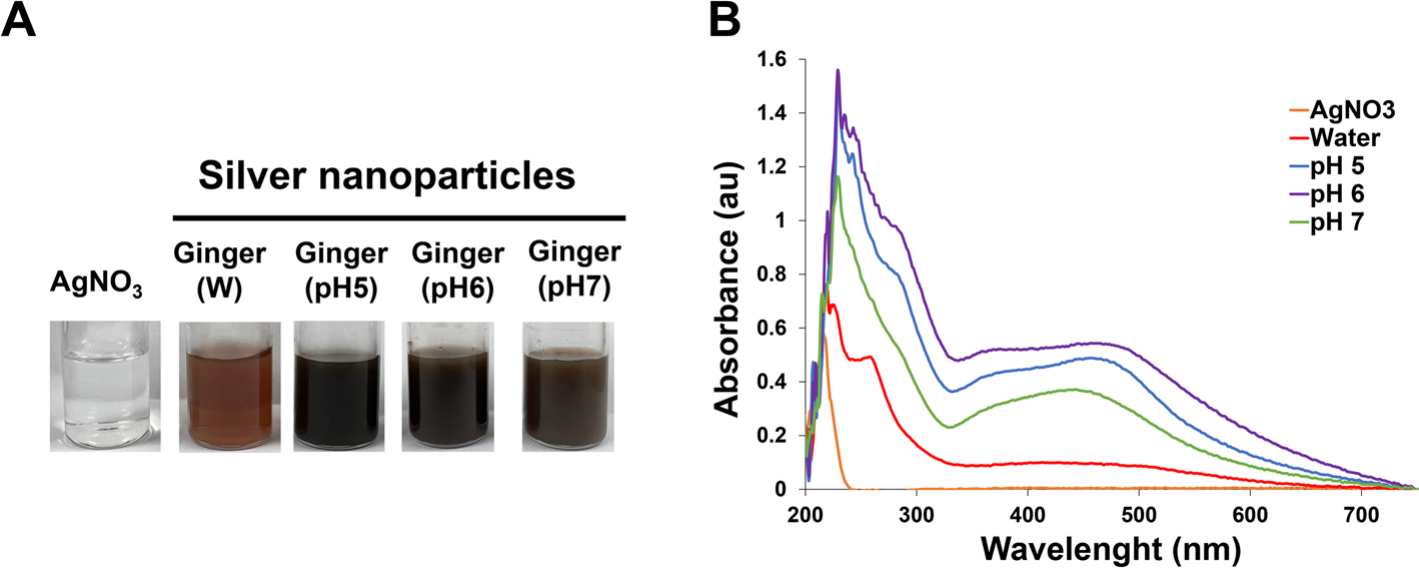
**A** Vials containing silver nitrate with colorless and AgNPs synthesized from ginger extract with water and various pH cosolvents. **B** UV–visible range spectra of the synthesized AgNPs using ginger extract in water and a 50:50 ethanol and ethyl acetate cosolvent at different pH levels

The addition of ginger plant extract to beakers containing an aqueous solution of silver nitrate leads to a rapid color change from yellow to reddish brown within the reaction time (Fig. 2A). This color transition occurs due to the excited surface plasmon vibrations in silver nanoparticles. When 1 mL of ginger extracts with varying pH values (5–7 with cosolvent 50:50 ethanol:ethyl acetate) is added in a concentration of 19 mL silver nitrate (2 mM), the color of the solution shifts from a pale light shade to yellowish brown, and eventually to colloidal brown. This change in color signifies the formation of silver nanoparticles within the aqueous silver nitrate solution. The analysis of the silver nanoparticles generated using 2 mM of silver nitrate and different pH levels of the ginger extract (ranging from pH 5 to 7) reveals the presence of a plasmon resonance band in the wavelength range of 400–500 nm [52].

Figure 2B illustrates that the wavelength of the ginger extract reaches its maximum value at 457 nm when the pH of the ginger extract is prepared to 6. The absorbance values exhibit slight shifts in other pHs, indicating changes in particle size [53]. Furthermore, the experimental findings establish that the absorption of light in the wavelength range of 400 to 500 nm is most pronounced at pH 6. This phenomenon can be attributed to the elevated concentration of phenolic components [54] and flavonoids present in the ginger extract at pH 6. As a result, ginger extracts prepared using ethanol and ethyl acetate at pH 6 hold significant value as reducing agents in the synthesis of GE-AgNPs.

#### Size and charge

**Fig. 3:**
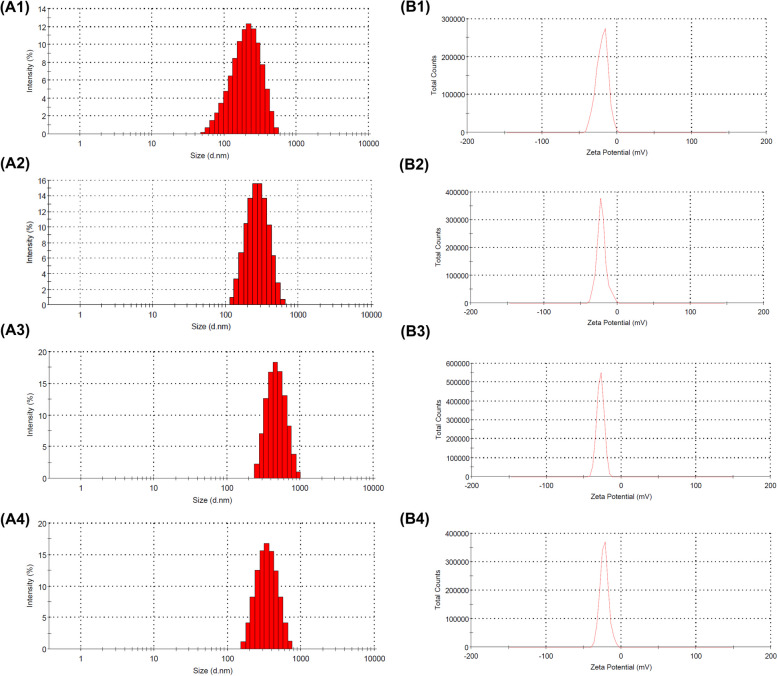
particle size histogram of silver nanoparticles with reducing agents of aqueous ginger extract (**A1**), cosolvent ginger extract in pH 5 (**A2**), pH 6 (**A3**), pH 7 (**A4**) and surface charge distribution with reducing agents of aqueous ginger extract (**B1**), cosolvent ginger extract in pH 5 (**B2**), pH 6 (**B3**), pH 7 (**B4**)

The average size, distribution, and propensity for aggregation of silver nanoparticles (AgNPs) were assessed using dynamic light scattering (DLS) in this study. The different pH values of the ethyl acetate:ethanol cosolvent or water, extracting ginger as the reducing agent, are performed. A comparative analysis of particle sizes indicated that AgNPs derived from the aqueous extract of ginger exhibited the smallest dimensions, whereas those dispersed in the cosolvent at pH 6 displayed the largest sizes, suggesting a potential inclination towards aggregation. The stability of nanoparticles is intricately linked to the pH value of the ginger extract. Figure 3 visually demonstrates the relationship between particle size and the acquisition of a negative charge, with smaller particles approaching a state of neutrality. Consequently, under pH 6 conditions, the particles manifest the most pronounced negative zeta potential value at -27.47 mV, ostensibly implying heightened stability and diminished proclivity toward aggregation compared to pH 5 and 7. Paradoxically, the observed particle size contradicts the anticipated trend based on the zeta potential, as the particles attain their maximum size at pH 6. Several potential explanations could account for this phenomenon [55–57]: 1) ionization of ginger extract components: the alteration of ionization states in specific phytochemicals present in the ginger extract around pH 6 may contribute to enhanced stabilization of silver ion reduction and the growth of larger particles. Alternatively, these changes may foster conditions conducive to aggregation, notwithstanding the higher zeta potential. 2) optimal reduction Environment: the reducing agents inherent in the ginger extract might exhibit maximum efficacy at pH 6, resulting in the swift formation of larger silver nanoparticles compared to conditions at pH 5 or pH 7. 3) kinetics of particle growth: the conditions at pH 6 may preferentially facilitate a specific kinetic pathway for nanoparticle formation, enabling the particles to attain a larger size before stabilization occurs.

#### Transmission electron microscopy (TEM) and energy-dispersive X-ray spectroscopy (EDS)

**Fig. 4:**
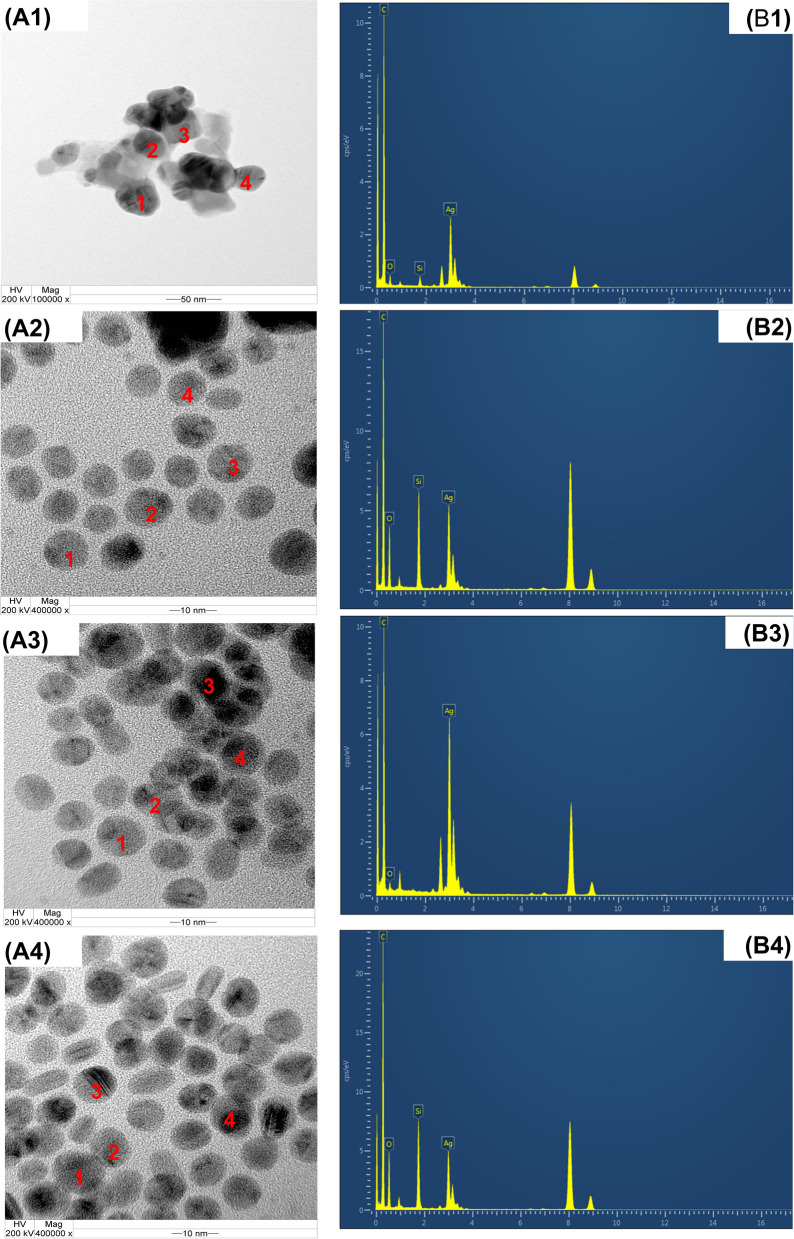
Transmission electron microscopy (TEM) of silver nanoparticles with reducing agents of aqueous ginger extract (**A1**), cosolvent ginger extract in pH 5 (**A2**), pH 6 (**A3**), pH 7 (**A4**) and EDS spectrum with reducing agents of aqueous ginger extract (**B1**), cosolvent ginger extract in pH 5 (**B2**), pH 6 (**B3**), pH 7 (**B4**)

Transmission electron microscopy (TEM) was employed to investigate the characteristics of the synthesized silver nanoparticles (AgNPs), including their size and morphology. The analysis involved the utilization of ginger extract in both water and cosolvent systems. The TEM examination unveiled that the AgNPs produced under these conditions displayed a predominantly spherical shape. The average size of the AgNPs s was determined to range between 28 and 105 nm, as evidenced by the TEM images (Fig. 4A).

In order to investigate the elemental distribution within the ginger extracts employed for the synthesis of silver nanoparticles (AgNPs), energy-dispersive X-ray spectroscopy (EDS) can be utilized. The EDS profile, depicted in Fig. 4B, provides insights into the elemental composition of ginger-capped AgNPs. The elements represented in Fig. 4B include Carbon, Oxygen, Silicon, and Silver. The presence of Silicon can be attributed to the underlying silicon base, as evident from the Si peak observed. Considering that ginger plants are utilized in the nanoparticle production process; the presence of carbon and oxygen is expected in the final product. The coexistence of oxygen peaks, along with the signals corresponding to silver, implies the protection of AgNPs through phenolate ion bonding. The robust signal of Ag atoms in the EDS profile serves as evidence of their crystalline nature. Furthermore, it is worth noting that the absorption of metallic silver nanocrystallites typically occurs around 3 keV, as supported by the optical absorption peak [58].

#### Fourier transform infrared spectroscopy (FTIR)

**Fig. 5:**
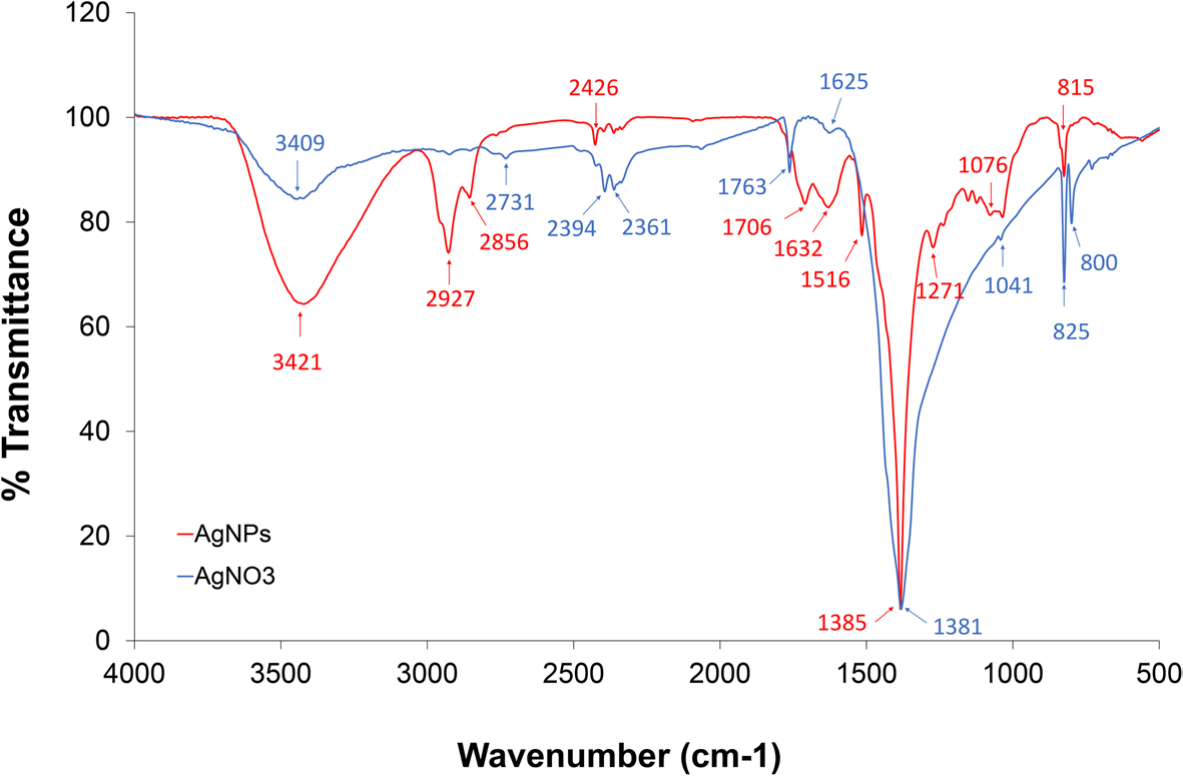
Fourier transform infrared spectra of silver nitrate (blue) and silver nanoparticles from ginger (red)

The biomolecules responsible for the efficient reduction, capping, and encapsulation of silver nanoparticles (AgNPs) were identified through Fourier transform infrared spectroscopy (FTIR). Similar peaks were observed in the FTIR spectra of both silver nitrate and AgNPs, albeit at slightly different wavelengths. This discovery provides compelling evidence for the presence of ginger extract as a natural component within the synthesized AgNPs. Additionally, the intricacy observed in the fingerprint region of the spectra indicates the presence of components originating from silver nitrate. Figure 5 illustrates representative FTIR spectra of both silver nitrate (used as a reference) and silver nanoparticles, with the assignment of IR bands based on relevant literature sources [59, 60]. Absorption peaks were observed in the FTIR spectra of the synthesized AgNPs at wavenumbers of 3421, 2927, 1632, 1385, 1076, and 815 cm^−1^ (Fig. 5). Particularly noteworthy is the identification of amide groups as the source of the bands observed at 3,400 cm^−1^ and within the range of 1,700 to 1,400 cm^−1^ in the AgNPs spectra. These findings strongly suggest an interaction between the silver nanoparticles and the predominant proteinaceous phytoconstituents present in ginger. Peaking at 3,409 cm^−1^, the stretching of N–H bonds in silver nitrate was observed in the higher wavenumber range (corresponding to lower frequencies). Following the synthesis of AgNPs, absorption peaks at 3,421 cm^−1^ and 1,076 cm^−1^, corresponding to the stretching bands of OH contributed by alcohols [61] and CN stretching vibration of the amine [62], respectively, originating from oligosaccharide residues in the plant extract, exhibited narrower profiles and migrated towards higher wavenumber regions. The peak at 2,927 cm^−1^ was attributed to the antisymmetric stretching of CH_2_ groups, which are abundantly found in lipids. Of significant relevance, the occurrence of the H_2_O bending (H–O-H) peak at 1,381 cm^−1^, representing the amide I vibration, was observed. Notably, the structure of proteins was modified by the interaction with the silver nanoparticles, as evidenced by the sharpening and shifting of this peak to a wavenumber of 1,385 cm^−1^. Therefore, these findings provide compelling evidence that proteins effectively encapsulated the silver nanoparticles derived from ginger extract, potentially facilitated by free amine groups or cysteine residues [63]. Furthermore, the identified wavenumbers revealed the presence of various functional groups within the silver nanoparticles synthesized using ginger extract, including alkyne, alkene, phenol, ether, and alkane. Remarkably, plants rich in alkaloids and polyphenolic compounds possess an extraordinary capacity to significantly reduce silver ions, resulting in the formation of silver nanoparticles, while also acting as highly effective stabilizing agents [64]. Moreover, The FTIR analysis of the ginger extract manifested spectral peaks at the wavenumbers 3287, 2924, 1634, 1513, 1076, and 992 cm-1 as mentioned in previous investigation [65]. Notably, these peaks exhibited slight shifts upon application in the synthesis of silver nanoparticles, a phenomenon discussed in prior study [66].

### In vitro cytotoxicity

**Fig. 6:**
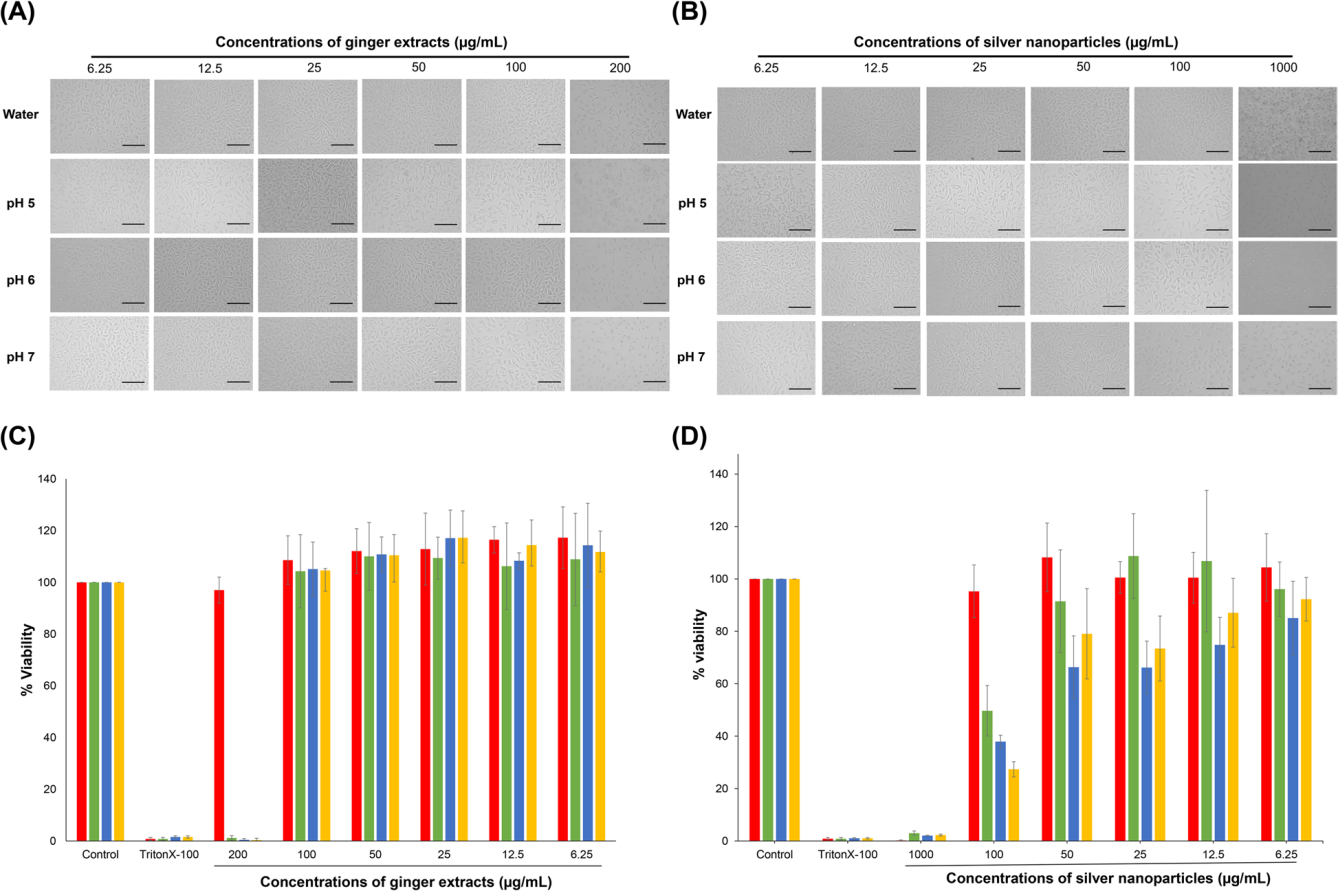
The morphology of L929 cell line: **A** co-cultured with ginger extracts and (**B**) silver nanoparticles. Cell viability of L929 Cells: **C** co-cultured with ginger extracts and (**D**) silver nanoparticles. Incubated for 24 h with aqueous ginger extract (red), cosolvent ginger extract at pH 5 (green), pH 6 (blue), pH 7 (orange), compared with untreated (Control) and toxic control (Triton-X-100). Scale bar: 250 µm

The viability of L929 cells was assessed using the MTT assay, in strict accordance with ISO 10993 guidelines, which dictate a minimum cell viability threshold of 80% [67]. Figure 6C illustrates that aqueous ginger extract does not induce cytotoxicity in fibroblast cells, even at concentrations as high as 200 µg/mL. Nevertheless, noteworthy alterations in cellular morphology, characterized by a rounded form, are discernible, as depicted in Fig. 6A. Conversely, the application of a cosolvent-assisted ginger extraction method exerts negligible influence on the cytotoxic response of fibroblast cells, even when exposed to the maximal concentration of 100 µg/mL. Notably, cellular morphology maintains its customary characteristics when contrasted against the control as evidenced in Fig. S3. Upon the synthesis of AgNPs by the aforementioned ginger extracts, a discernable escalation in cytotoxicity towards fibroblast cells is ascertained. Figure 6D illustrates the outcomes of this evaluation, revealing that AgNPs obtained from ginger extract in water demonstrated superior biocompatibility, with cell viability consistently exceeding 80% at concentrations below 100 μg/mL. In contrast, AgNPs synthesized with cosolvents (50 ethyl acetate: 50 ethanol) exhibited marginally heightened cytotoxicity. Furthermore, the biocompatibility of AgNPs was influenced by the pH of the cosolvent preparation, with AgNPs synthesized at pH 5 being safe at a concentration of 68.38 ± 10.82 μg/mL, while those prepared at pH 7 and pH 6 were safe at concentrations of 39.32 ± 12.52 μg/mL and 17.52 ± 7.00 μg/mL, respectively. The concurrence of these findings with the maximum silver concentration detected via EDS analysis, as depicted in Fig. 4. In all instances, the cellular morphology of L*929* cells subjected to assessment with GE-AgNPs utilizing a cosolvent, at a concentration of *50* µg/mL, remained devoid of conspicuous alterations, as evidenced by the findings presented in Fig. 6B, relative to the control group. These observations serve to underscore the safety profile of GE-AgNPs across various pH levels of cosolvent extraction, provided they are judiciously dimensioned as elucidated earlier.

### Molecular docking

**Fig. 7:**
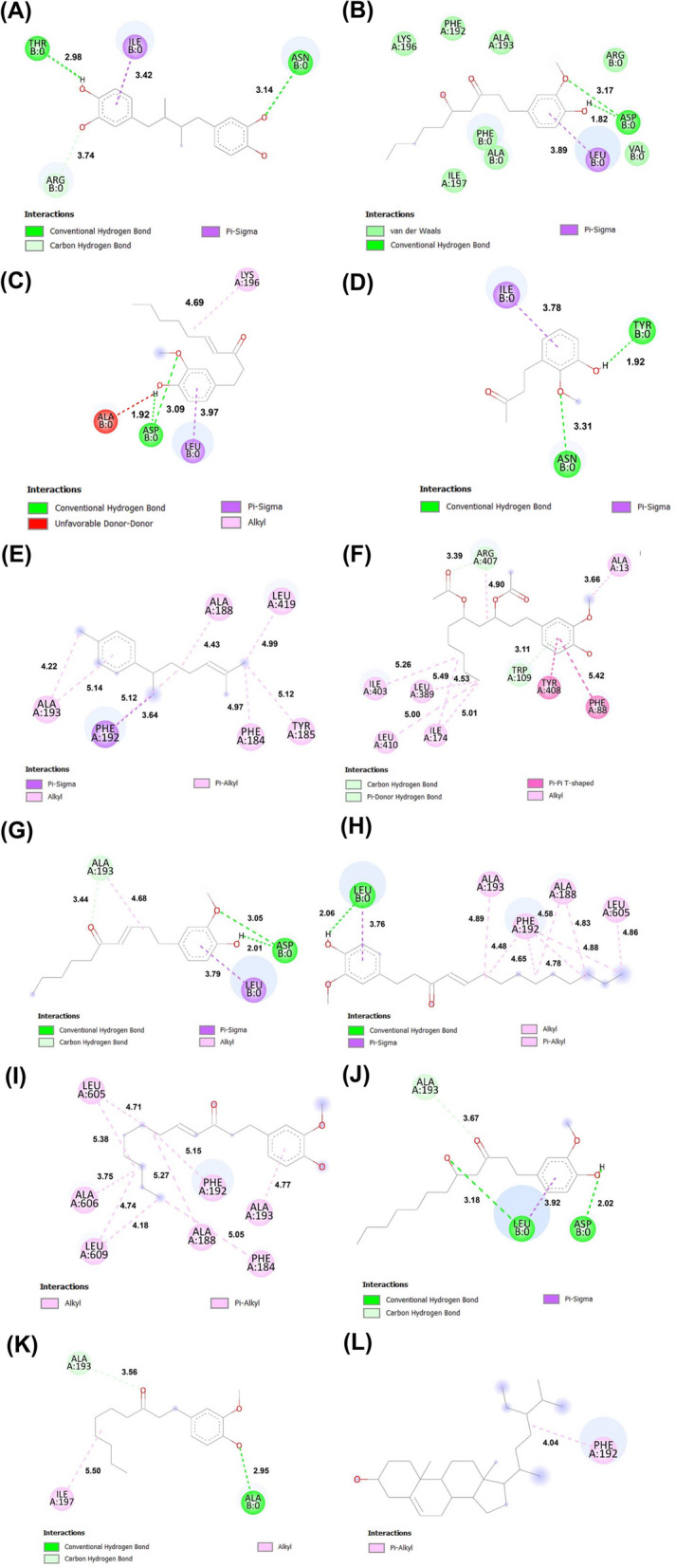
Molecular docking conformation of (**A**) positive control as nordihydroguaiaretic acid, and ginger extracts containing (**B**) 6-gingerol, (**C**) 6-shogaol, (**D**) butan-2-one, 4-(3-hydroxy-2-methoxyphenyl)-, (**E**) Alpha-curcumene, (**F**) diacetoxy-6-gingerdiol, (**G**) 6-isoshogaol, (**H**) 3-decanone,1-(4-hydroxy-3-methoxyphenyl)-, (**I**) 8-shogaol, (**J**) (S)-8-gingerol, (**K**) 1-(4-hydroxy-3-methoxyphenyl)tetradec-4-en-3-one, and (**L**) clionasterol at active site of LOX

The process of molecular docking was executed on the lipoxygenase enzyme employing the evaluated ginger extracts. To achieve this aim, the three-dimensional structure of lipoxygenase (PDB ID: 7LAF) was employed as a reference. The docking scores for the various poses were assessed, only retaining those with a total energy lower than -9.0, -6.0, and -4.0 kcal mol^−1^ in ArgusLab, Vina, and Autodock, respectively, as depicted in Table 4. The comprehensive set of docking scores, encompassing the standard ligand (nordihydroguaiaretic acid), and those compounds extracted from ginger, is available in Table 4. In the context of the lipoxygenase molecular docking, Clionasterol exhibited the most favorable docking score, resulting in an overall calculated binding energy of -8.9 kcal mol^−1^. A comparative analysis revealed that the ligand nordihydroguaiaretic acid exhibited a lower docking score than the other compounds, with a total energy of -8.0 kcal mol^−1^. Figure 7 elucidates the primary interactions between clionasterol and the specific amino acid residues within the binding site of lipoxygenase. While clionasterol exhibits a diminished binding energy in comparison to the standard ligand, it is noteworthy that the frequency of hydrogen bond occurrences is notably reduced. This observation suggests a diminished binding stability of the compound, rendering clionasterol more susceptible to dissociation as contrasted with the standard ligand. In this context, it becomes apparent that the molecular structure of *6*-gingerol facilitates the formation of hydrogen bonds at two distinct ligand positions: one engaging ASP with a spatial separation of *1.82* Å and *3.17* Å, and another involving a Pi-Sigma interaction with the amino acid LEU. Conversely, the standard ligand establishes hydrogen bonds at two specific positions, entailing distances of *2.98* Å and *3.14* Å with the amino acid THR and ASN, along with a singular Pi-Sigma interaction with the amino acid ILE. As a result, it becomes evident that 6-gingerol manifests a more robust binding affinity towards LOX [68] relative to the standard ligand. This finding aligns cohesively with the discerned outcomes of the Structure–Activity Relationship (SAR) analysis as shown in Table S3, wherein alpha-zingiberene, sesquiphellandrene, and beta-bisabolene, by virtue of unfavorable bonding interactions, do not demonstrate appreciable binding with the LOX protein. Furthermore, the investigation also reveals that compounds such as butan-*2*-one, *4-(3-*hydroxy*-2-*methoxyphenyl)-, alpha-curcumene, diacetoxy-*6*-gingerdiol, *6*-isoshogaol, *8*-shogaol, and *1-(4*-hydroxy-*3*-methoxyphenyl)tetradec-*4*-en-*3*-one are capable of inhibiting LOX activity through a mechanism without unfavorable bonding. The diverse nature of these prominent compounds contributes substantively to the augmentation of LOX inhibition efficiency. Therefore, the synthesis of silver nanoparticles using ginger extract is crucial in enabling the aforementioned particles to inhibit inflammation through the LOX mechanism.

**Table 4:** Molecular docking data of positive control as nordihydroguaiaretic acid, and ginger compounds against lipoxygenase

Compounds	Contents (%)		Binding affinity (kcal/mol) of ligands to LOX		
	Water	Cosolvent	Arguslab	Vina	Autodock
Nordihydroguaiaretic acid			-11.5195	-8.0	-5.12
6-gingerol	27.69	9.83	-11.1077	-6.4	-4.11
6-shogaol	11.56	9.11	-12.2919	-6.3	-4.51
Butan-2-one, 4-(3-hydroxy-2-methoxyphenyl)-	9.26	4.43	-9.0293	-6.2	-4.08
Alpha-zingiberene	6.29	7.35	-12.0969	-7.9	-5.83
Alpha-curcumene	6.26	3.57	-13.141	-7.6	-5.45
Sesquiphellandrene	3.73	4.22	-12.1881	-6.7	-5.98
Beta-bisabolene	3.60	4.24	-12.8127	-7.7	-5.56
Diacetoxy-6-gingerdiol	1.92	2.05	-10.2678	-7.0	-2.97
6-isoshogaol	0.93	9.11	12.7547	-6.8	-5.46
3-decanone,1-(4-hydroxy-3-methoxyphenyl)-	0.63	1.11	-11.7984	-6.1	-4.68
8-shogaol	1.35	2.57	-12.7358	-7.0	-5.33
(S)-8-gingerol	1.28	1.31	-12.2436	-6.5	-4.25
1-(4-hydroxy-3-methoxyphenyl)tetradec-4-en-3-one	1.08	2.87	-11.2954	-6.9	-4.95
Clionasterol	0.83	1.37	-14.1923	-8.9	-8.61

### In-vitro lipoxygenase inhibitory activity

**Fig. 8:**
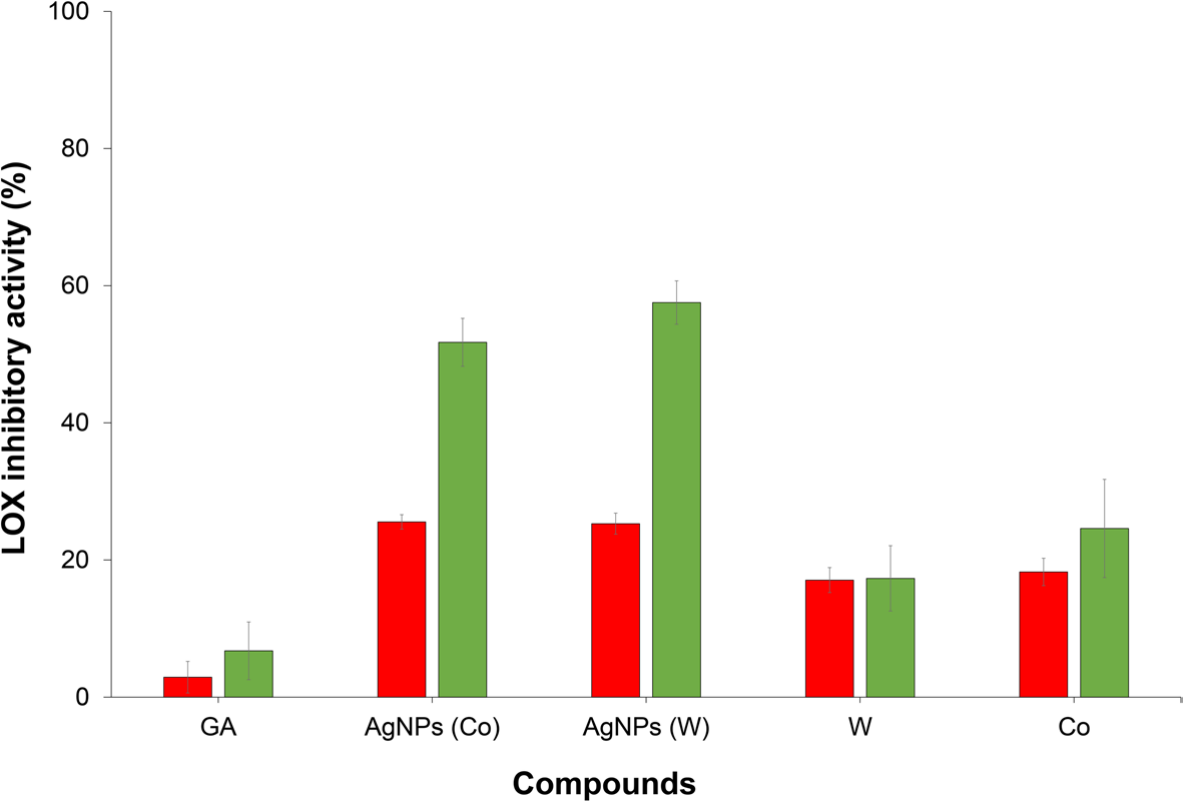
linoleic acid was employed as the substrate to investigate the potential inhibition of lipoxygenase (LOX) activity. Gallic acid (GA), utilized as a positive control, alongside silver nanoparticles synthesized from ginger extracted using co-solvent represented by AgNPs(Co), silver nanoparticles synthesized from ginger extracted using water depicted by AgNPs(W), ginger extracted using water (W), and ginger extracted using co-solvent (Co) were tested at concentrations of 10 g/mL (red bars) and 100 µg/mL (green bars). Notably, all tested compounds exhibited inhibitory effects on the enzyme activity. Error bars in the graph signify the standard deviation from the mean, and each bar represents the average result derived from three independent experimental trials

The findings of anti-inflammatory efficacy experiments revealed notable differences when comparing the effects of water extract ginger, co-solvent extract ginger (ethyl acetate: ethanol), and silver nanoparticles synthesized using these extracts, with a particular emphasis on their performance at higher concentrations. Notably, all investigated substances exhibited enhanced anti-inflammatory properties in inhibiting lipoxygenase (LOX) activity at elevated concentrations. Specifically, silver nanoparticles synthesized from water extract ginger exhibited remarkable LOX inhibitory effects at concentrations of *10* and *100* µg/mL, resulting in inhibitory percentages of *25.29* ± *1.53* and *57.53* ± *3.16*, respectively. In contrast, water extract ginger, when applied at equivalent concentrations, displayed lower LOX inhibitory activities, with inhibitory percentages of *17.07* ± *1.83* and *17.32* ± *4.77*, respectively, as indicated in Fig. 8. Similarly, silver nanoparticles synthesized from co-solvent extract ginger at concentrations of *10* and *100* µg/mL consistently demonstrated substantial LOX inhibition, yielding inhibitory percentages of *25.55* ± *1.06* and *51.74* ± *3.50*, respectively. In contrast, co-solvent extract ginger, under the same concentration conditions, exhibited lower LOX inhibitory activities, resulting in inhibitory percentages of *18.25* ± *2.00* and *24.60* ± *7.16*, respectively. Notably, all tested samples outperformed gallic acid, employed as the positive control, in terms of LOX inhibition across the entire concentration range. These results provide compelling evidence that silver nanoparticles synthesized from water extract ginger and co-solvent extract ginger possess superior LOX inhibitory properties compared to direct ginger extract.

## Conclusion

The synthesis of AgNPs was conducted utilizing a conventional chemical method, with the utilization of *Zingiber officinale* extract as a reducing agent. The AgNPs underwent a thorough analysis that encompassed several physical, structural, and morphological properties. These properties included size, shape, and dispersity, which were evaluated using techniques such as UV–Vis spectroscopy, Zeta potential measurements, Fourier-transform infrared spectroscopy (FTIR), transmission electron microscopy (TEM), energy-dispersive X-ray spectroscopy (EDS) examinations. The GE-AgNPs exhibited significant antioxidant properties, as indicated by the results of radical scavenging experiments. The ginger extracts obtained by the use of ethyl acetate and ethanol, with a pH of 6 and a concentration of 6.83 ± 0.47 µg/mL, shown significantly improved ability to scavenge free radicals when compared to well-known antioxidants as Trolox and Ascorbic acid. Significantly, the ginger extracts demonstrated anti-inflammatory properties that can be linked to the suppression of lipoxygenase (LOX) activity. The study demonstrated that silver nanoparticles synthesized from water extract ginger and co-solvent extract ginger exhibit significantly enhanced lipoxygenase (LOX) inhibitory properties, especially at higher concentrations, outperforming direct ginger extract and the positive control, gallic acid. These findings suggest the potential therapeutic value of these nanoparticles for managing inflammation. In addition, the biocompatibility of GE-AgNPs was found to be up to 17.52 ± 7.00 µg/mL. Furthermore, experimental confirmation through molecular docking [69] revealed that *6*-gingerol exhibited a stronger binding affinity towards LOX. This aligns with the Structure–Activity Relationship analysis, emphasizing its potential to enhance LOX inhibition efficiency. This underscores the importance of synthesizing silver nanoparticles using ginger extract for inhibiting inflammation through the LOX mechanism. The combined results of this study highlight the significant bioactive, biocompatible, and anti-inflammatory properties of GE-AgNPs, suggesting their potential use as a valuable addition to cosmetic products, with the recommended concentration being 10 µg/mL to maintain their free radical scavenging, anti-inflammatory, and safety properties.

### Supplementary Information

**Additional file 1:** **Supplementary Table 1.** Gas chromatography-tandem mass spectrometry (GC-MS/MS) of *Zingiber officinale* extracted with 50% ethanol and 50% ethyl acetate mixture. **Supplementary Table 2.** Gas chromatography-tandem mass spectrometry (GC-MS/MS) of *Zingiber officinale* extracted with water. **Supplementary Table 3.** Active compounds and bioactivities of *Zingiber officinale* extracted with water (GW) and cosolvent between 50% ethanol and 50% ethyl acetate mixture (GE). **Supplementary Table 4.** PASS analysis of 6-gingerol. **Supplementary Table 5.** PASS analysis of 6-shogaol. **Supplementary Table 6.** PASS analysis of Zingiberine. **Supplementary Table 7.** PASS analysis of butan-2-one, 4-(3-hydroxy-2-methoxyphenyl). **Supplementary Table 8.** PASS analysis of beta-bisabolene. **Supplementary Table 9.** PASS analysis of sesquiphellandrene. **Supplementary Table 10.** PASS analysis of alpha-curcumene. **Supplementary Table 11.** PASS analysis of 1-(4-hydroxy-3-methoxyphenyl)tetradec-4-en-3-one. **Supplementary Table 12.** PASS analysis of 8-shogaol. **Supplementary Table 13.** PASS analysis of diacetoxy-6-gingerdiol. **Supplementary Table 14.** PASS analysis of 6-isoshogaol. **Supplementary Table 15.** PASS analysis of clionasterol. **Supplementary Table 16.** PASS analysis of (S)-8-gingerol. **Supplementary Table 17.** PASS analysis of 3-decanone,1-(4-hydroxy-3-methoxyphenyl).

**Additional file 2:** **Figure S1.** GC-MS/MS chromatogram graph of water extract of Zingiber officinale.

**Additional file 3:** **Figure S2.** GC-MS/MS chromatogram graph of 50% ethanol and 50% ethyl acetate mixture of Zingiber officinale.

**Additional file 4:** **Figure S3.** Cytotoxicity of L929 cells exposed to (A) complete medium (negative control) and (B) Triton-X-100 (positive control). Scale bar: 250 µm.

## Data Availability

The data that support the findings of this study are available in supplementary file.
