# Supplementary material for: Green-synthesized silver nanoparticles from Zingiber officinale extract: antioxidant potential, biocompatibility, anti-LOX properties, and in silico analysis
Source: BMC Complement Med Ther. 2024 Feb 13;24:84. doi: 10.1186/s12906-024-04381-w (PMC10863109; doi:10.1186/s12906-024-04381-w)
Supplement: Supplementary file 1 — Additional file 1: Supplementary Table 1. Gas chromatography-tandem mass spectrometry (GC-MS/MS) of Zingiber officinale extracted with 50% ethanol and 50% ethyl acetate mixture. Supplementary Table 2. Gas chromatography-tandem mass spectrometry (GC-MS/MS) of Zingiber officinale extracted with water. Supplementary Table 3. Active compounds and bioactivities of Zingiber officinale extracted with water (GW) and cosolvent between 50% ethanol and 50% ethyl acetate mixture (GE). Supplementary Table 4. PASS analysis of 6-gingerol. Supplementary Table 5. PASS analysis of 6-shogaol. Supplementary Table 6. PASS analysis of Zingiberine. Supplementary Table 7. PASS analysis of butan-2-one, 4-(3-hydroxy-2-methoxyphenyl). Supplementary Table 8. PASS analysis of beta-bisabolene. Supplementary Table 9. PASS analysis of sesquiphellandrene. Supplementary Table 10. PASS analysis of alpha-curcumene. Supplementary Table 11. PASS analysis of 1-(4-hydroxy-3-methoxyphenyl)tetradec-4-en-3-one. Supplementary Table 12. PASS analysis of 8-shogaol. Supplementary Table 13. PASS analysis of diacetoxy-6-gingerdiol. Supplementary Table 14. PASS analysis of 6-isoshogaol. Supplementary Table 15. PASS analysis of clionasterol. Supplementary Table 16. PASS analysis of (S)-8-gingerol. Supplementary Table 17. PASS analysis of 3-decanone,1-(4-hydroxy-3-methoxyphenyl). [file 12906_2024_4381_MOESM1_ESM.docx]

**Supplementary table 1** Gas chromatography-tandem mass spectrometry (GC-MS/MS) of *Zingiber officinale* extracted with 50% ethanol and 50% ethyl acetate mixture.

| **RT** | **Compound Name** | **Formula** | **Component Area** | **Match Factor** |
| --- | --- | --- | --- | --- |
| 8.9169 | 5-Hepten-2-one,6-methyl- | C_8_H_14_O | 2301417.8 | 92.3 |
| 9.4357 | Octanal | C_8_H_16_O | 7655135.8 | 97.1 |
| 10.3022 | EUCALYPTOL(1,8-CINEOLE) | C_10_H_18_O | 7101123.6 | 97.7 |
| 12.7198 | Linalool | C_10_H_18_O | 5140517.2 | 97.2 |
| 14.6935 | Bicyclo[2.2.1]heptan-2-ol,1,7,7-trimethyl-,(1S-endo)- | C_10_H_18_O | 19598358.2 | 98.4 |
| 15.0626 | 3-Cyclohexen-1-ol,4-methyl-1-(1-methylethyl)- | C_10_H_18_O | 2261023.1 | 93.8 |
| 15.4905 | L-.alpha.-Terpineol | C_10_H_18_O | 17569502 | 98.2 |
| 15.9826 | Decanal | C_10_H_20_O | 16460235.6 | 99.1 |
| 16.6779 | 6-Octen-1-ol,3,7-dimethyl- | C_10_H_20_O | 12817762.5 | 97.7 |
| 17.2876 | 2-Caren-4-ol | C_10_H_16_O | 1639304.9 | 90.6 |
| 17.4321 | Geraniol | C_10_H_18_O | 26002295 | 98.3 |
| 17.86 | 2,6-Octadienal,3,7-dimethyl- | C_10_H_16_O | 2537646 | 93.3 |
| 18.2504 | Bicyclo[2.2.1]heptan-2-ol,1,7,7-trimethyl-,acetate,(1S-endo)- | C_12_H_20_O_2_ | 2628205.6 | 95.4 |
| 18.5125 | 2-Undecanone | C_11_H_22_O | 4932159.3 | 96.7 |
| 19.0367 | 2-Methoxy-4-vinylphenol | C_9_H_10_O_2_ | 8612369.6 | 93.8 |
| 19.978 | (1S,4S,4aS)-1-Isopropyl-4,7-dimethyl-1,2,3,4,4a,5-hexahydronaphthalene | C_15_H_24_ | 872092.7 | 90.2 |
| 20.1278 | 6-Octen-1-ol,3,7-dimethyl-,acetate | C_12_H_22_O_2_ | 5018119.5 | 95.2 |
| 20.3632 | 1,2,4-Metheno-1H-indene,octahydro-1,7adimethyl-5-(1-methylethyl)-,[1S-(1.alpha.,2.alpha.,3a.beta.,4.alpha.,5.alpha.,7a.beta.,8S*)]- | C_15_H_24_ | 5570248.8 | 91 |
| 20.668 | Copaene | C_15_H_24_ | 10216666.4 | 97 |
| 20.9301 | Geranyl acetate | C_12_H_20_O_2_ | 16674487.2 | 97.8 |
| 21.1066 | 2,4-DIISOPROPENYL-1-METHYL-1-VINYLCYCLOHEXANE | C_15_H_24_ | 11414899.1 | 95 |
| 21.481 | (1S,5S)-2-Methyl-5-((R)-6-methylhept-5-en-2-yl)bicyclo[3.1.0]hex-2-ene | C_15_H_24_ | 5104374.8 | 93.2 |
| 21.7752 | Tricyclo[4.4.0.0(2,7)]decane,1-methyl-3-methylene-8-(1-methylethyl)-,stereoisomer | C_15_H_24_ | 3237184.6 | 93.1 |
| 22.0319 | (1R,2S,6S,7S,8S)-8-Isopropyl-1-methyl-3-methylenetricyclo[4.4.0.02,7]decane-rel- | C_15_H_24_ | 2207962.3 | 94.1 |
| 22.1603 | Cyclohexane,1-ethenyl-1-methyl-2-(1-methylethenyl)-4-(1-methylethylidene)- | C_15_H_24_ | 12093533.9 | 96.3 |
| 22.5401 | Naphthalene,decahydro-4a-methyl-1-methylene-7-(1-methylethylidene)-,(4aR-trans)- | C_15_H_24_ | 2887840.7 | 92.5 |
| 22.7808 | cis-.beta.-Farnesene | C_15_H_24_ | 5861568.7 | 94.4 |
| 23.2354 | Naphthalene,1,2,3,4,4a,5,6,8a-octahydro-7-methyl-4-methylene-1-(1-methylethyl)-,(1.alpha.,4a.alpha.,8a.alpha.)- | C_15_H_24_ | 16801302.4 | 96.3 |
| 23.3317 | (1R,3aR,4aR,8aR)-1,4,4,6-Tetramethyl-1,2,3,3a,4,4a,7,8-octahydrocyclopenta[1,4]cyclobuta[1,2]benzene | C_15_H_24_ | 19826545.4 | 95.3 |
| 23.4333 | Benzene,1-(1,5-dimethyl-4-hexenyl)-4-methyl- | C_15_H_22_ | 114085568.1 | 97.7 |
| 23.6366 | 1H-Cyclopropa[a]naphthalene,decahydro-1,1,3atrimethyl-7-methylene-,[1aS-(1a.alpha.,3a.alpha.,7a.beta.,7b.alpha.)]- | C_15_H_24_ | 26072029.3 | 96 |
| 23.7917 | 1,3-Cyclohexadiene,5-(1,5-dimethyl-4-hexenyl)-2-methyl-,[S-(R*,S*)]- | C_15_H_24_ | 234742356 | 97.5 |
| 24.0752 | .beta.-Bisabolene | C_15_H_24_ | 135273055.1 | 93.6 |
| 24.241 | (2S,4aR,8aR)-4a,8-Dimethyl-2-(prop-1-en-2-yl)-1,2,3,4,4a,5,6,8a-octahydronaphthalene | C_15_H_24_ | 8656914.7 | 94.4 |
| 24.4496 | Cyclohexene,3-(1,5-dimethyl-4-hexenyl)-6-methylene-,[S-(R*,S*)]- | C_15_H_24_ | 134898137.5 | 98.5 |
| 24.5993 | (Z)-1-Methyl-4-(6-methylhept-5-en-2-ylidene)cyclohex-1-ene | C_15_H_24_ | 8589695.4 | 91.9 |
| 25.0005 | Cyclohexanemethanol,4-ethenyl-.alpha.,.alpha.,4-trimethyl-3-(1-methylethenyl)-,[1R-(1.alpha.,3.alpha.,4.beta.)]- | C_15_H_26_O | 8799101.3 | 97.1 |
| 25.3535 | 1,6,10-Dodecatrien-3-ol,3,7,11-trimethyl- | C_15_H_26_O | 24720255.3 | 97.3 |
| 25.5888 | 4-(1-Hydroxyallyl)-2-methoxyphenol | C_10_H_12_O_3_ | 11585786.7 | 95.4 |
| 25.9526 | trans-Sesquisabinene hydrate | C_15_H_26_O | 6666136.1 | 96.1 |
| 26.4981 | (1R,4R)-1-methyl-4-(6-Methylhept-5-en-2-yl)cyclohex-2-enol | C_15_H_26_O | 8014092.2 | 93.7 |
| 26.6051 | 2-Naphthalenemethanol,1,2,3,4,4a,5,6,7-octahydro-.alpha.,.alpha.,4a,8-tetramethyl-,(2Rcis)- | C_15_H_26_O | 5521121.3 | 95.5 |
| 27.3058 | Butan-2-one,4-(3-hydroxy-2-methoxyphenyl)- | C_11_H_14_O_3_ | 141305962.4 | 95.5 |
| 29.5308 | Phenol,5-(1,5-dimethyl-4-hexenyl)-2-methyl-,(R)- | C_15_H_22_O | 6995941.1 | 91.3 |
| 30.7771 | (1R,4aR,7R,8aR)-7-(2-Hydroxypropan-2-yl)-1,4adimethyldecahydronaphthalen-1-ol | C_15_H_28_O_2_ | 6253079.4 | 92.7 |
| 33.874 | n-Hexadecanoic acid | C_16_H_32_O_2_ | 8798750.7 | 93.8 |
| 34.3768 | Hexadecanoic acid,ethyl ester | C_18_H_36_O_2_ | 14748164.3 | 97.5 |
| 35.9867 | 1-(4-Hydroxy-3-methoxyphenyl)oct-4-en-3-one | C_15_H_20_O_3_ | 6843158.7 | 90.9 |
| 37.4095 | Linoleic acid ethyl ester | C_20_H_36_O_2_ | 9172685.6 | 96.5 |
| 37.5218 | Ethyl Oleate | C_20_H_38_O_2_ | 4679561.1 | 93.6 |
| 37.6662 | 5-Hydroxy-1-(4-hydroxy-3-methoxyphenyl)octan-3-one | C_15_H_22_O_4_ | 9291772.9 | 91.9 |
| 38.415 | (E)-1-(4-Hydroxy-3-methoxyphenyl)dec-3-en-5-one | C_17_H_24_O_3_ | 62381064.1 | 94.9 |
| 38.5755 | 3-Decanone,1-(4-hydroxy-3-methoxyphenyl)- | C_17_H_26_O_3_ | 35565709 | 96.1 |
| 39.7255 | 1-(4-Hydroxy-3-methoxyphenyl)dec-4-en-3-one | C_17_H_24_O_3_ | 290776695.6 | 97.7 |
| 41.3354 | 5-Hydroxy-1-(4-hydroxy-3-methoxyphenyl)decan-3-one | C_17_H_26_O_4_ | 314021413.5 | 97.3 |
| 42.9775 | 1-(4-Hydroxy-3-methoxyphenyl)dodec-4-en-3-one | C_19_H_28_O_3_ | 81958393 | 95.1 |
| 43.3679 | (3R,5S)-1-(4-Hydroxy-3-methoxyphenyl)decane-3,5-diyl diacetate | C_21_H_32_O_6_ | 65510516.8 | 94.9 |
| 44.3842 | (E)-1-(4-hydroxy-3-methoxyphenyl)dec-1-ene-3,5-dione | C_17_H_22_O_4_ | 16881750.4 | 90.8 |
| 44.5072 | 5-Hydroxy-1-(4-hydroxy-3-methoxyphenyl)dodecan-3-one | C_19_H_30_O_4_ | 41736142.2 | 94.8 |
| 46.1332 | 1-(4-Hydroxy-3-methoxyphenyl)tetradec-4-en-3-one | C_21_H_32_O_3_ | 91592835.7 | 97.5 |
| 51.2252 | (E)-4-(2-(2-(2,6-Dimethylhepta-1,5-dien-1-yl)-6-pentyl-1,3-dioxan-4-yl)ethyl)-2-methoxyphenol | C_27_H_42_O_4_ | 26063621.1 | 92.5 |
| 52.7442 | Campesterol | C_28_H_48_O | 7851006.4 | 92.3 |
| 53.1561 | Stigmasta-5,22-dien-3-ol,(3.beta.,22E)- | C_29_H_48_O | 15051849.4 | 92.6 |
| 53.8675 | .gamma.-Sitosterol | C_29_H_50_O | 43898410.4 | 97.3 |

**Supplementary table 2** Gas chromatography-tandem mass spectrometry (GC-MS/MS) of *Zingiber officinale* extracted with water.

| **RT** | **Compound Name** | **Other name** | **CAS#** | **Formula** | **Component Area** | **Match Factor** |
| --- | --- | --- | --- | --- | --- | --- |
| 23.3958 | Benzene, 1-(1,5-dimethyl-4-hexenyl)-4-methyl- | alpha-Curcumene | 644-30-4 | C_15_H_22_ | 9979953.6 | 97.4 |
| 23.7114 | 1,3-Cyclohexadiene, 5-(1,5-dimethyl-4-hexenyl)-2-methyl-, [S-(R*,S*)]- | a-zingiberene | 495-60-3 | C_15_H_24_ | 10033201.4 | 96 |
| 24.0323 | .beta.-Bisabolene | n/a | 495-61-4 | C_15_H_24_ | 5740974.5 | 95.1 |
| 24.3907 | Cyclohexene, 3-(1,5-dimethyl-4-hexenyl)-6-methylene-, [S-(R*,S*)]- | Sesquiphellandrene | 20307-83-9 | C_15_H_24_ | 5953472.2 | 97.2 |
| 27.2309 | Butan-2-one, 4-(3-hydroxy-2-methoxyphenyl)- | n/a | 303187-89-5 | C_11_H_14_O_3_ | 14772450.9 | 97.1 |
| 39.5757 | 1-(4-Hydroxy-3-methoxyphenyl)dec-4-en-3-one | 6-Shogaol | 555-66-8 | C_17_H_24_O_3_ | 18431640.1 | 97.6 |
| 41.1535 | 5-Hydroxy-1-(4-hydroxy-3-methoxyphenyl)decan-3-one | rac-[6]-Gingerol | 39886-76-5 | C_17_H_26_O_4_ | 44160547.1 | 97.9 |
| 43.3198 | (3R,5S)-1-(4-Hydroxy-3-methoxyphenyl)decane-3,5-diyl diacetate | Diacetoxy-6-gingerdiol | 143615-75-2 | C_21_H_32_O_6_ | 3058293 | 92.4 |

**Supplementary table 3** Active compounds and bioactivities of *Zingiber officinale* extracted with water (GW) and cosolvent between 50% ethanol and 50% ethyl acetate mixture (GE).

| **Compounds** | **GW**  **% content** | **GE**  **% content** | **Antioxidant** | **Anti-LOX** |
| --- | --- | --- | --- | --- |
| 6-gingerol | 27.69 | 9.83 | n/a | ✓ |
| 6-Shogaol | 11.56 | 9.11 | ✓ | ✓ |
| Butan-2-one, 4-(3-hydroxy-2-methoxyphenyl)- | 9.26 | 4.43 | n/a | ✓ |
| a-zingiberene | 6.29 | 7.35 | n/a | n/a |
| alpha-Curcumene | 6.26 | 3.57 | n/a | ✓ |
| Sesquiphellandrene | 3.73 | 4.22 | n/a | n/a |
| .beta.-Bisabolene | 3.60 | 4.24 | n/a | n/a |
| Diacetoxy-6-gingerdiol | 1.92 | 2.05 | n/a | ✓ |
| 6-Isoshogaol | n/a | 9.11 | ✓ | ✓ |
| 3-Decanone,1-(4-hydroxy-3-methoxyphenyl)- | n/a | 1.11 | n/a | ✓ |
| 8-Shogaol | n/a | 2.57 | ✓ | ✓ |
| S-8-Gingerol | n/a | 1.31 | n/a | ✓ |
| 1-(4-Hydroxy-3-methoxyphenyl)tetradec-4-en-3-one | n/a | 2.87 | ✓ | ✓ |
| Clionasterol | n/a | 1.37 | n/a | ✓ |

**Supplementary table 4** PASS analysis of 6-gingerol

| **PA** | **Pi** | **Activities** |
| --- | --- | --- |
| 0,960 | 0,003 | 5 Hydroxytryptamine release stimulant |
| 0,911 | 0,003 | Linoleate diol synthase inhibitor |
| 0,860 | 0,021 | CYP2C12 substrate |
| 0,817 | 0,010 | Feruloyl esterase inhibitor |
| 0,803 | 0,007 | UDP-glucuronosyltransferase substrate |
| 0,817 | 0,027 | Ubiquinol-cytochrome-c reductase inhibitor |
| 0,772 | 0,004 | Preneoplastic conditions treatment |
| 0,771 | 0,004 | Steroid N-acetylglucosaminyltransferase inhibitor |
| 0,762 | 0,007 | Macrophage colony stimulating factor agonist |
| 0,757 | 0,004 | Mycothiol-S-conjugate amidase inhibitor |
| 0,758 | 0,008 | Fibrinolytic |
| 0,765 | 0,027 | Gluconate 2-dehydrogenase (acceptor) inhibitor |
| 0,757 | 0,027 | Polyporopepsin inhibitor |
| 0,730 | 0,007 | Vasodilator, peripheral |
| 0,707 | 0,005 | Beta-carotene 15,15'-monooxygenase inhibitor |
| 0,735 | 0,035 | Chymosin inhibitor |
| 0,735 | 0,035 | Acrocylindropepsin inhibitor |
| 0,735 | 0,035 | Saccharopepsin inhibitor |
| 0,730 | 0,034 | Chlordecone reductase inhibitor |
| 0,717 | 0,020 | GST A substrate |
| 0,740 | 0,051 | Aspulvinone dimethylallyltransferase inhibitor |

**Supplementary table 5** PASS analysis of 6-shogaol

| **Pa** | **Pi** | **Activities** |
| --- | --- | --- |
| 0,927 | 0,003 | Linoleate diol synthase inhibitor |
| 0,873 | 0,002 | Preneoplastic conditions treatment |
| 0,876 | 0,007 | 5 Hydroxytryptamine release stimulant |
| 0,863 | 0,002 | MMP9 expression inhibitor |
| 0,855 | 0,002 | Beta-carotene 15,15'-monooxygenase inhibitor |
| 0,851 | 0,003 | Antimutagenic |
| 0,849 | 0,009 | Mucomembranous protector |
| 0,834 | 0,002 | Steroid N-acetylglucosaminyltransferase inhibitor |
| 0,842 | 0,011 | Antieczematic |
| 0,828 | 0,006 | JAK2 expression inhibitor |
| 0,812 | 0,011 | HIF1A expression inhibitor |
| 0,819 | 0,027 | Ubiquinol-cytochrome-c reductase inhibitor |
| 0,820 | 0,028 | Aspulvinone dimethylallyltransferase inhibitor |
| 0,803 | 0,013 | Mucositis treatment |
| 0,772 | 0,002 | Vanillyl-alcohol oxidase inhibitor |
| 0,772 | 0,009 | UDP-glucuronosyltransferase substrate |
| 0,767 | 0,004 | HMOX1 expression enhancer |
| 0,791 | 0,038 | CYP2C12 substrate |
| 0,754 | 0,004 | TNF expression inhibitor |
| 0,772 | 0,025 | Chlordecone reductase inhibitor |
| 0,757 | 0,016 | TP53 expression enhancer |
| 0,769 | 0,029 | CYP2J substrate |
| 0,743 | 0,003 | Free radical scavenger |
| 0,759 | 0,028 | Gluconate 2-dehydrogenase (acceptor) inhibitor |
| 0,748 | 0,022 | CYP2J2 substrate |
| 0,728 | 0,005 | Reductant |
| 0,734 | 0,018 | Feruloyl esterase inhibitor |
| 0,730 | 0,019 | GST A substrate |
| 0,707 | 0,004 | GST M substrate |
| 0,717 | 0,014 | Antiinflammatory |
| 0,705 | 0,003 | Peroxidase substrate |
| 0,715 | 0,017 | Fibrinolytic |
| 0,711 | 0,014 | Apoptosis agonist |

**Supplementary table 6** PASS analysis of Zingiberine

| **Pa** | **Pi** | **Activities** |
| --- | --- | --- |
| 0,836 | 0,003 | Carminative |
| 0,842 | 0,010 | Mucomembranous protector |
| 0,825 | 0,001 | Retinol dehydrogenase inhibitor |
| 0,819 | 0,027 | Ubiquinol-cytochrome-c reductase inhibitor |
| 0,798 | 0,018 | Alkenylglycerophosphocholine hydrolase inhibitor |
| 0,794 | 0,020 | Antieczematic |
| 0,758 | 0,008 | Fibrinolytic |
| 0,773 | 0,028 | CYP2J substrate |
| 0,749 | 0,014 | Protein-disulfide reductase (glutathione) inhibitor |
| 0,736 | 0,005 | Prenyl-diphosphatase inhibitor |
| 0,743 | 0,015 | Alkylacetylglycerophosphatase inhibitor |
| 0,728 | 0,003 | Vitamin-K-epoxide reductase (warfarin-insensitive) inhibitor |
| 0,731 | 0,022 | Acylcarnitine hydrolase inhibitor |
| 0,711 | 0,002 | Antiviral (Rhinovirus) |
| 0,703 | 0,006 | Undecaprenyl-phosphate mannosyltransferase inhibitor |

**Supplementary table 7** PASS analysis of butan-2-one, 4-(3-hydroxy-2-methoxyphenyl)

| **Pa** | **Pi** | **Activities** |
| --- | --- | --- |
| 0,925 | 0,003 | Gluconate 2-dehydrogenase (acceptor) inhibitor |
| 0,923 | 0,005 | Aspulvinone dimethylallyltransferase inhibitor |
| 0,868 | 0,009 | Chlordecone reductase inhibitor |
| 0,867 | 0,008 | 5 Hydroxytryptamine release stimulant |
| 0,842 | 0,004 | Platelet derived growth factor receptor kinase inhibitor |
| 0,829 | 0,006 | JAK2 expression inhibitor |
| 0,833 | 0,011 | Mucomembranous protector |
| 0,823 | 0,005 | Linoleate diol synthase inhibitor |
| 0,812 | 0,004 | Fibrinolytic |
| 0,810 | 0,011 | Feruloyl esterase inhibitor |
| 0,811 | 0,029 | Ubiquinol-cytochrome-c reductase inhibitor |
| 0,811 | 0,034 | CYP2C12 substrate |
| 0,796 | 0,020 | Antiseborrheic |
| 0,759 | 0,004 | Antipyretic |
| 0,754 | 0,004 | MMP9 expression inhibitor |
| 0,754 | 0,005 | Preneoplastic conditions treatment |
| 0,788 | 0,039 | Membrane integrity agonist |
| 0,738 | 0,004 | Steroid N-acetylglucosaminyltransferase inhibitor |
| 0,729 | 0,004 | Beta glucuronidase inhibitor |
| 0,724 | 0,004 | Endothelial growth factor antagonist |
| 0,710 | 0,006 | Carminative |
| 0,722 | 0,027 | Taurine dehydrogenase inhibitor |
| 0,722 | 0,048 | Testosterone 17beta-dehydrogenase (NADP+) inhibitor |

**Supplementary table 8** PASS analysis of beta-bisabolene

| **Pa** | **Pi** | **Activities** |
| --- | --- | --- |
| 0,908 | 0,001 | Retinol dehydrogenase inhibitor |
| 0,904 | 0,001 | Myc inhibitor |
| 0,899 | 0,004 | Apoptosis agonist |
| 0,895 | 0,002 | Carminative |
| 0,868 | 0,008 | Antieczematic |
| 0,856 | 0,006 | Antineoplastic |
| 0,798 | 0,001 | Alpha-pinene-oxide decyclase inhibitor |
| 0,789 | 0,010 | CYP2C substrate |
| 0,787 | 0,022 | Mucomembranous protector |
| 0,762 | 0,005 | CYP2C19 substrate |
| 0,771 | 0,028 | CYP2J substrate |
| 0,723 | 0,003 | Chemoprotective |
| 0,720 | 0,005 | Prenyl-diphosphatase inhibitor |
| 0,726 | 0,013 | Antiinflammatory |
| 0,722 | 0,014 | Immunosuppressant |
| 0,708 | 0,005 | Antineoplastic (breast cancer) |
| 0,707 | 0,004 | Transcription factor NF kappa B stimulant |
| 0,707 | 0,004 | Transcription factor stimulant |

**Supplementary table 9** PASS analysis of sesquiphellandrene

| **Pa** | **Pi** | **Activities** |
| --- | --- | --- |
| 0,904 | 0,005 | Antieczematic |
| 0,827 | 0,009 | Antineoplastic |
| 0,805 | 0,017 | Mucomembranous protector |
| 0,789 | 0,019 | Alkenylglycerophosphocholine hydrolase inhibitor |
| 0,766 | 0,002 | Retinol dehydrogenase inhibitor |
| 0,760 | 0,012 | Prostaglandin-E2 9-reductase inhibitor |
| 0,750 | 0,004 | Antipsoriatic |
| 0,742 | 0,015 | Protein-disulfide reductase (glutathione) inhibitor |
| 0,720 | 0,004 | Vitamin-K-epoxide reductase (warfarin-insensitive) inhibitor |
| 0,732 | 0,016 | Alkylacetylglycerophosphatase inhibitor |
| 0,720 | 0,005 | Prenyl-diphosphatase inhibitor |
| 0,708 | 0,006 | Carminative |
| 0,718 | 0,024 | Acylcarnitine hydrolase inhibitor |
| 0,702 | 0,016 | Immunosuppressant |
| 0,726 | 0,042 | CYP2J substrate |
| 0,704 | 0,067 | Ubiquinol-cytochrome-c reductase inhibitor |

**Supplementary table 10** PASS analysis of alpha-curcumene

| **Pa** | **Pi** | **Activities** |
| --- | --- | --- |
| 0,942 | 0,004 | Mucomembranous protector |
| 0,876 | 0,010 | Ubiquinol-cytochrome-c reductase inhibitor |
| 0,872 | 0,007 | Antieczematic |
| 0,849 | 0,011 | CYP2J substrate |
| 0,827 | 0,005 | Phosphatidylcholine-retinol O-acyltransferase inhibitor |
| 0,816 | 0,003 | Prenyl-diphosphatase inhibitor |
| 0,825 | 0,014 | Alkenylglycerophosphocholine hydrolase inhibitor |
| 0,815 | 0,004 | All-trans-retinyl-palmitate hydrolase inhibitor |
| 0,791 | 0,010 | Protein-disulfide reductase (glutathione) inhibitor |
| 0,783 | 0,004 | Carminative |
| 0,781 | 0,005 | Fatty-acyl-CoA synthase inhibitor |
| 0,776 | 0,004 | Undecaprenyl-phosphate mannosyltransferase inhibitor |
| 0,791 | 0,028 | Testosterone 17beta-dehydrogenase (NADP+) inhibitor |
| 0,772 | 0,014 | Glutamyl endopeptidase II inhibitor |
| 0,792 | 0,036 | Aspulvinone dimethylallyltransferase inhibitor |
| 0,768 | 0,012 | Alkylacetylglycerophosphatase inhibitor |
| 0,757 | 0,003 | Vitamin-K-epoxide reductase (warfarin-insensitive) inhibitor |
| 0,756 | 0,002 | Plastoquinol-plastocyanin reductase inhibitor |
| 0,754 | 0,008 | Linoleate diol synthase inhibitor |
| 0,739 | 0,004 | Beta-carotene 15,15'-monooxygenase inhibitor |
| 0,748 | 0,020 | Acylcarnitine hydrolase inhibitor |
| 0,728 | 0,002 | Retinol dehydrogenase inhibitor |
| 0,743 | 0,023 | CYP2J2 substrate |
| 0,723 | 0,003 | BRAF expression inhibitor |
| 0,728 | 0,014 | Fibrinolytic |
| 0,715 | 0,004 | Gastrin inhibitor |
| 0,717 | 0,007 | Cholesterol antagonist |
| 0,715 | 0,006 | Adenomatous polyposis treatment |
| 0,722 | 0,048 | CDP-glycerol glycerophosphotransferase inhibitor |

**Supplementary table 11** PASS analysis of 1-(4-hydroxy-3-methoxyphenyl)tetradec-4-en-3-one

| **Pa** | **Pi** | **Activities** |
| --- | --- | --- |
| 0,927 | 0,003 | Linoleate diol synthase inhibitor |
| 0,873 | 0,002 | Preneoplastic conditions treatment |
| 0,876 | 0,007 | 5 Hydroxytryptamine release stimulant |
| 0,863 | 0,002 | MMP9 expression inhibitor |
| 0,855 | 0,002 | Beta-carotene 15,15'-monooxygenase inhibitor |
| 0,851 | 0,003 | Antimutagenic |
| 0,849 | 0,009 | Mucomembranous protector |
| 0,834 | 0,002 | Steroid N-acetylglucosaminyltransferase inhibitor |
| 0,842 | 0,011 | Antieczematic |
| 0,828 | 0,006 | JAK2 expression inhibitor |
| 0,812 | 0,011 | HIF1A expression inhibitor |
| 0,819 | 0,027 | Ubiquinol-cytochrome-c reductase inhibitor |
| 0,820 | 0,028 | Aspulvinone dimethylallyltransferase inhibitor |
| 0,803 | 0,013 | Mucositis treatment |
| 0,772 | 0,002 | Vanillyl-alcohol oxidase inhibitor |
| 0,772 | 0,009 | UDP-glucuronosyltransferase substrate |
| 0,767 | 0,004 | HMOX1 expression enhancer |
| 0,791 | 0,038 | CYP2C12 substrate |
| 0,754 | 0,004 | TNF expression inhibitor |
| 0,772 | 0,025 | Chlordecone reductase inhibitor |
| 0,757 | 0,016 | TP53 expression enhancer |
| 0,769 | 0,029 | CYP2J substrate |
| 0,743 | 0,003 | Free radical scavenger |
| 0,759 | 0,028 | Gluconate 2-dehydrogenase (acceptor) inhibitor |
| 0,748 | 0,022 | CYP2J2 substrate |
| 0,728 | 0,005 | Reductant |
| 0,734 | 0,018 | Feruloyl esterase inhibitor |
| 0,730 | 0,019 | GST A substrate |
| 0,707 | 0,004 | GST M substrate |
| 0,717 | 0,014 | Antiinflammatory |
| 0,705 | 0,003 | Peroxidase substrate |
| 0,715 | 0,017 | Fibrinolytic |
| 0,711 | 0,014 | Apoptosis agonist |

**Supplementary table 12** PASS analysis of 8-shogaol

| **Pa** | **Pi** | **Activities** |
| --- | --- | --- |
| 0,927 | 0,003 | Linoleate diol synthase inhibitor |
| 0,873 | 0,002 | Preneoplastic conditions treatment |
| 0,876 | 0,007 | 5 Hydroxytryptamine release stimulant |
| 0,863 | 0,002 | MMP9 expression inhibitor |
| 0,855 | 0,002 | Beta-carotene 15,15'-monooxygenase inhibitor |
| 0,851 | 0,003 | Antimutagenic |
| 0,849 | 0,009 | Mucomembranous protector |
| 0,834 | 0,002 | Steroid N-acetylglucosaminyltransferase inhibitor |
| 0,842 | 0,011 | Antieczematic |
| 0,828 | 0,006 | JAK2 expression inhibitor |
| 0,812 | 0,011 | HIF1A expression inhibitor |
| 0,819 | 0,027 | Ubiquinol-cytochrome-c reductase inhibitor |
| 0,820 | 0,028 | Aspulvinone dimethylallyltransferase inhibitor |
| 0,803 | 0,013 | Mucositis treatment |
| 0,772 | 0,002 | Vanillyl-alcohol oxidase inhibitor |
| 0,772 | 0,009 | UDP-glucuronosyltransferase substrate |
| 0,767 | 0,004 | HMOX1 expression enhancer |
| 0,791 | 0,038 | CYP2C12 substrate |
| 0,754 | 0,004 | TNF expression inhibitor |
| 0,772 | 0,025 | Chlordecone reductase inhibitor |
| 0,757 | 0,016 | TP53 expression enhancer |
| 0,769 | 0,029 | CYP2J substrate |
| 0,743 | 0,003 | Free radical scavenger |
| 0,759 | 0,028 | Gluconate 2-dehydrogenase (acceptor) inhibitor |
| 0,748 | 0,022 | CYP2J2 substrate |
| 0,728 | 0,005 | Reductant |
| 0,734 | 0,018 | Feruloyl esterase inhibitor |
| 0,730 | 0,019 | GST A substrate |
| 0,707 | 0,004 | GST M substrate |
| 0,717 | 0,014 | Antiinflammatory |
| 0,705 | 0,003 | Peroxidase substrate |
| 0,715 | 0,017 | Fibrinolytic |
| 0,711 | 0,014 | Apoptosis agonist |

**Supplementary table 13** PASS analysis of diacetoxy-6-gingerdiol

| **Pa** | **Pi** | **Activities** |
| --- | --- | --- |
| 0,814 | 0,005 | Lipid metabolism regulator |
| 0,809 | 0,013 | 5 Hydroxytryptamine release stimulant |
| 0,799 | 0,006 | Linoleate diol synthase inhibitor |
| 0,770 | 0,009 | Hypolipemic |
| 0,789 | 0,037 | Ubiquinol-cytochrome-c reductase inhibitor |
| 0,749 | 0,005 | Preneoplastic conditions treatment |
| 0,784 | 0,040 | CYP2C12 substrate |
| 0,739 | 0,005 | MMP9 expression inhibitor |
| 0,748 | 0,017 | Feruloyl esterase inhibitor |
| 0,727 | 0,005 | TNF expression inhibitor |
| 0,749 | 0,030 | Chlordecone reductase inhibitor |
| 0,717 | 0,007 | Antihypercholesterolemic |
| 0,711 | 0,005 | Steroid N-acetylglucosaminyltransferase inhibitor |
| 0,751 | 0,048 | Aspulvinone dimethylallyltransferase inhibitor |
| 0,738 | 0,040 | Mucomembranous protector |
| 0,723 | 0,030 | Membrane permeability inhibitor |
| 0,703 | 0,021 | Fibrinolytic |
| 0,709 | 0,042 | Acrocylindropepsin inhibitor |
| 0,709 | 0,042 | Saccharopepsin inhibitor |
| 0,709 | 0,042 | Chymosin inhibitor |

**Supplementary table 14** PASS analysis of 6-isoshogaol

| **Pa** | **Pi** | **Activities** |
| --- | --- | --- |
| 0,901 | 0,003 | Linoleate diol synthase inhibitor |
| 0,899 | 0,002 | Preneoplastic conditions treatment |
| 0,851 | 0,009 | Mucomembranous protector |
| 0,834 | 0,002 | Steroid N-acetylglucosaminyltransferase inhibitor |
| 0,828 | 0,006 | JAK2 expression inhibitor |
| 0,824 | 0,003 | Beta-carotene 15,15'-monooxygenase inhibitor |
| 0,807 | 0,004 | Antimutagenic |
| 0,803 | 0,003 | MMP9 expression inhibitor |
| 0,811 | 0,011 | HIF1A expression inhibitor |
| 0,819 | 0,027 | Ubiquinol-cytochrome-c reductase inhibitor |
| 0,820 | 0,028 | Aspulvinone dimethylallyltransferase inhibitor |
| 0,803 | 0,013 | Mucositis treatment |
| 0,801 | 0,014 | 5 Hydroxytryptamine release stimulant |
| 0,793 | 0,020 | Antieczematic |
| 0,791 | 0,038 | CYP2C12 substrate |
| 0,755 | 0,004 | TNF expression inhibitor |
| 0,754 | 0,006 | Antihypercholesterolemic |
| 0,772 | 0,025 | Chlordecone reductase inhibitor |
| 0,759 | 0,028 | Gluconate 2-dehydrogenase (acceptor) inhibitor |
| 0,735 | 0,018 | GST A substrate |
| 0,723 | 0,006 | HMOX1 expression enhancer |
| 0,734 | 0,018 | Feruloyl esterase inhibitor |
| 0,714 | 0,004 | GST M substrate |
| 0,709 | 0,004 | GST P substrate |
| 0,706 | 0,004 | Free radical scavenger |
| 0,709 | 0,014 | Apoptosis agonist |
| 0,719 | 0,044 | CYP2J substrate |

**Supplementary table 15** PASS analysis of clionasterol

| **Pa** | **Pi** | **Activities** |
| --- | --- | --- |
| 0,965 | 0,001 | DELTA14-sterol reductase inhibitor |
| 0,960 | 0,002 | Antihypercholesterolemic |
| 0,959 | 0,002 | Prostaglandin-E2 9-reductase inhibitor |
| 0,957 | 0,001 | Cholesterol antagonist |
| 0,952 | 0,002 | Alkenylglycerophosphocholine hydrolase inhibitor |
| 0,945 | 0,002 | Alkylacetylglycerophosphatase inhibitor |
| 0,928 | 0,003 | Acylcarnitine hydrolase inhibitor |
| 0,924 | 0,004 | Hypolipemic |
| 0,924 | 0,004 | Testosterone 17beta-dehydrogenase (NADP+) inhibitor |
| 0,917 | 0,001 | UGT1A4 substrate |
| 0,909 | 0,005 | CYP2C substrate |
| 0,903 | 0,003 | UGT1A substrate |
| 0,888 | 0,002 | UGT2B substrate |
| 0,889 | 0,003 | UDP-glucuronosyltransferase substrate |
| 0,886 | 0,002 | UGT2B1 substrate |
| 0,886 | 0,003 | Oxidoreductase inhibitor |
| 0,881 | 0,004 | Anesthetic general |
| 0,882 | 0,007 | CYP3A4 substrate |
| 0,869 | 0,001 | CYP4B substrate |
| 0,868 | 0,004 | CYP3A4 inducer |
| 0,866 | 0,004 | CYP3A inducer |
| 0,859 | 0,001 | CYP4B1 substrate |
| 0,858 | 0,002 | Adenomatous polyposis treatment |
| 0,865 | 0,008 | CYP3A substrate |
| 0,856 | 0,004 | Dextranase inhibitor |
| 0,855 | 0,004 | Linoleate diol synthase inhibitor |
| 0,848 | 0,003 | CYP2C11 substrate |
| 0,849 | 0,006 | Respiratory analeptic |
| 0,847 | 0,003 | Cholestanetriol 26-monooxygenase inhibitor |
| 0,835 | 0,003 | Alkenylglycerophosphoethanolamine hydrolase inhibitor |
| 0,832 | 0,004 | Sulfotransferase substrate |
| 0,831 | 0,003 | Chemopreventive |
| 0,818 | 0,002 | Peroxidase substrate |
| 0,816 | 0,002 | CYP17 inhibitor |
| 0,815 | 0,004 | Hepatoprotectant |
| 0,824 | 0,015 | CYP2J substrate |
| 0,806 | 0,005 | Caspase 3 stimulant |
| 0,804 | 0,003 | Proliferative diseases treatment |
| 0,804 | 0,005 | 27-Hydroxycholesterol 7alpha-monooxygenase inhibitor |
| 0,801 | 0,006 | CYP3A5 substrate |
| 0,789 | 0,001 | Cholesterol oxidase inhibitor |
| 0,800 | 0,013 | CYP2J2 substrate |
| 0,796 | 0,010 | Protein-disulfide reductase (glutathione) inhibitor |
| 0,788 | 0,002 | N-(long-chain-acyl)ethanolamine deacylase inhibitor |
| 0,788 | 0,005 | Glucan endo-1,3-beta-D-glucosidase inhibitor |
| 0,780 | 0,002 | Peptidoglycan glycosyltransferase inhibitor |
| 0,778 | 0,000 | Cholesterol synthesis inhibitor |
| 0,796 | 0,019 | Antieczematic |
| 0,769 | 0,003 | Trans-1,2-dihydrobenzene-1,2-diol dehydrogenase inhibitor |
| 0,762 | 0,005 | Antipruritic |
| 0,759 | 0,005 | Dermatologic |
| 0,762 | 0,009 | Immunosuppressant |
| 0,749 | 0,008 | Glyceryl-ether monooxygenase inhibitor |
| 0,745 | 0,005 | HMOX1 expression enhancer |
| 0,744 | 0,005 | CYP2C19 substrate |
| 0,736 | 0,002 | UGT2B4 substrate |
| 0,729 | 0,004 | Alcohol O-acetyltransferase inhibitor |
| 0,725 | 0,003 | UGT1A8 substrate |
| 0,717 | 0,001 | Cycloartenol synthase inhibitor |
| 0,716 | 0,002 | UGT2B7 substrate |
| 0,718 | 0,005 | Bone diseases treatment |
| 0,716 | 0,003 | Plasmanylethanolamine desaturase inhibitor |
| 0,744 | 0,037 | Mucomembranous protector |
| 0,753 | 0,047 | CYP2C12 substrate |
| 0,708 | 0,006 | Prostate disorders treatment |
| 0,706 | 0,005 | Antiosteoporotic |
| 0,703 | 0,013 | Lipoprotein lipase inhibitor |

**Supplementary table 16** PASS analysis of (S)-8-gingerol

| **Pa** | **Pi** | **Activities** |
| --- | --- | --- |
| 0,960 | 0,003 | 5 Hydroxytryptamine release stimulant |
| 0,911 | 0,003 | Linoleate diol synthase inhibitor |
| 0,860 | 0,021 | CYP2C12 substrate |
| 0,817 | 0,010 | Feruloyl esterase inhibitor |
| 0,803 | 0,007 | UDP-glucuronosyltransferase substrate |
| 0,817 | 0,027 | Ubiquinol-cytochrome-c reductase inhibitor |
| 0,772 | 0,004 | Preneoplastic conditions treatment |
| 0,771 | 0,004 | Steroid N-acetylglucosaminyltransferase inhibitor |
| 0,762 | 0,007 | Macrophage colony stimulating factor agonist |
| 0,757 | 0,004 | Mycothiol-S-conjugate amidase inhibitor |
| 0,758 | 0,008 | Fibrinolytic |
| 0,765 | 0,027 | Gluconate 2-dehydrogenase (acceptor) inhibitor |
| 0,757 | 0,027 | Polyporopepsin inhibitor |
| 0,730 | 0,007 | Vasodilator, peripheral |
| 0,707 | 0,005 | Beta-carotene 15,15'-monooxygenase inhibitor |
| 0,735 | 0,035 | Chymosin inhibitor |
| 0,735 | 0,035 | Acrocylindropepsin inhibitor |
| 0,735 | 0,035 | Saccharopepsin inhibitor |
| 0,730 | 0,034 | Chlordecone reductase inhibitor |
| 0,717 | 0,020 | GST A substrate |
| 0,740 | 0,051 | Aspulvinone dimethylallyltransferase inhibitor |

**Supplementary table 17** PASS analysis of 3-decanone,1-(4-hydroxy-3-methoxyphenyl)

| **Pa** | **Pi** | **Activities** |
| --- | --- | --- |
| 0,915 | 0,004 | 5 Hydroxytryptamine release stimulant |
| 0,861 | 0,002 | MMP9 expression inhibitor |
| 0,864 | 0,013 | Ubiquinol-cytochrome-c reductase inhibitor |
| 0,830 | 0,005 | Linoleate diol synthase inhibitor |
| 0,784 | 0,004 | Preneoplastic conditions treatment |
| 0,789 | 0,012 | TP53 expression enhancer |
| 0,790 | 0,039 | CYP2C12 substrate |
| 0,736 | 0,004 | Steroid N-acetylglucosaminyltransferase inhibitor |
| 0,730 | 0,004 | Mycothiol-S-conjugate amidase inhibitor |
| 0,734 | 0,013 | Fibrinolytic |
| 0,736 | 0,038 | Gluconate 2-dehydrogenase (acceptor) inhibitor |
| 0,740 | 0,051 | Aspulvinone dimethylallyltransferase inhibitor |
| 0,719 | 0,035 | Polyporopepsin inhibitor |
