# Supplementary figures and images for: Green-synthesized silver nanoparticles from Zingiber officinale extract: antioxidant potential, biocompatibility, anti-LOX properties, and in silico analysis
Source: BMC Complement Med Ther. 2024 Feb 13;24:84. doi: 10.1186/s12906-024-04381-w (PMC10863109; doi:10.1186/s12906-024-04381-w)

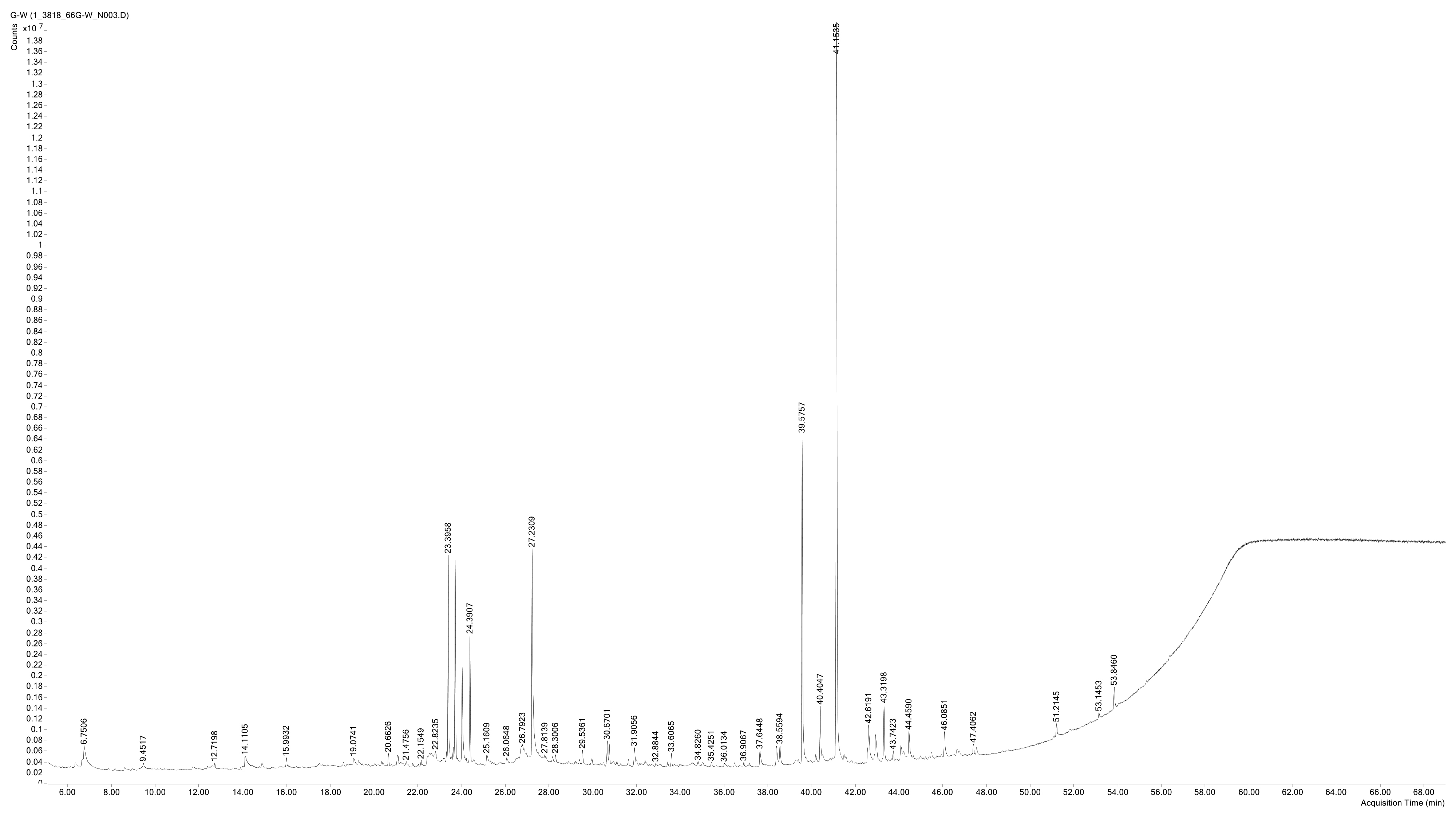

Supplement: Supplementary file 2 — Additional file 2: Figure S1. GC-MS/MS chromatogram graph of water extract of Zingiber officinale. [file 12906_2024_4381_MOESM2_ESM.jpg]

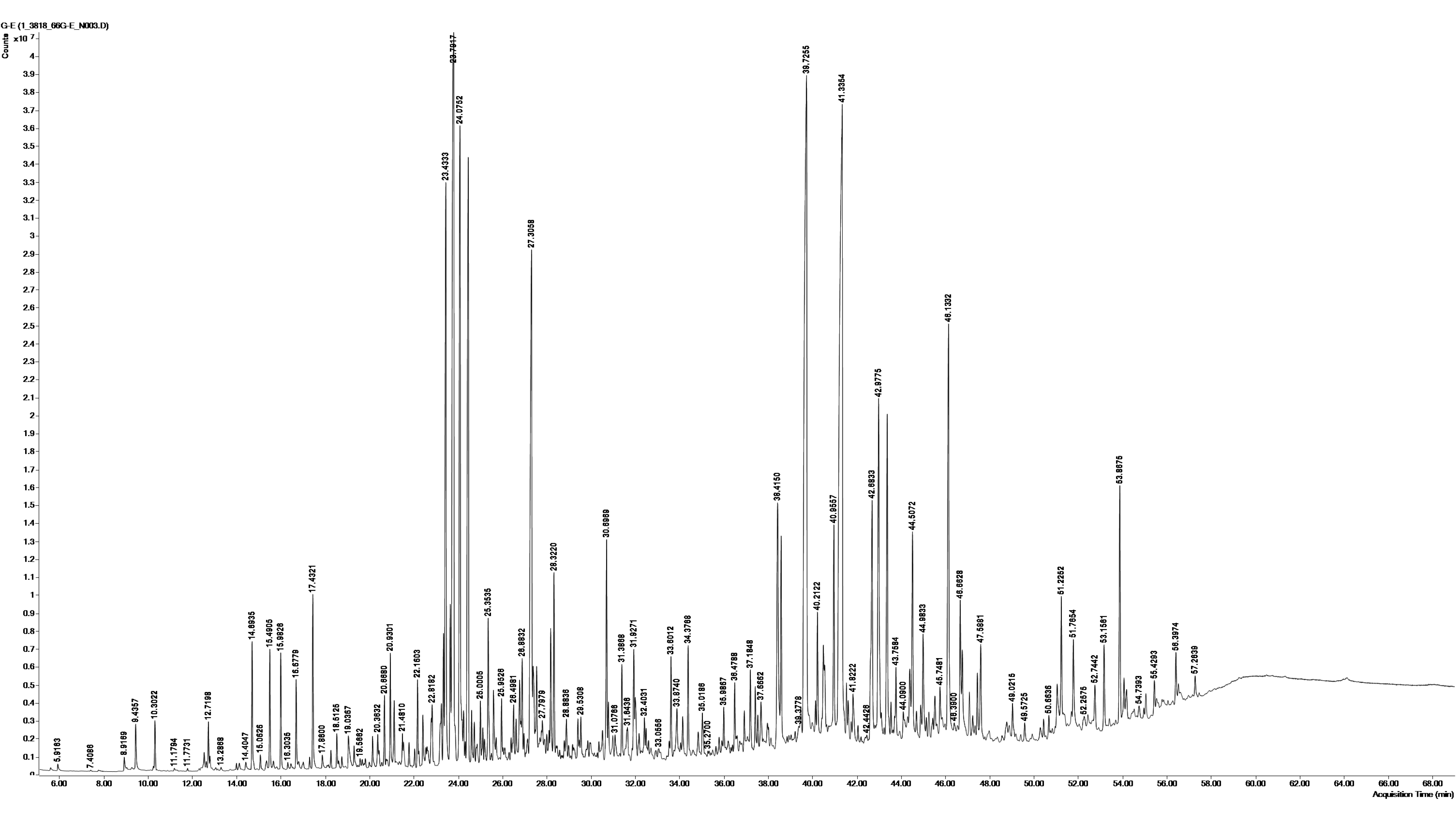

Supplement: Supplementary file 3 — Additional file 3: Figure S2. GC-MS/MS chromatogram graph of 50% ethanol and 50% ethyl acetate mixture of Zingiber officinale. [file 12906_2024_4381_MOESM3_ESM.jpg]

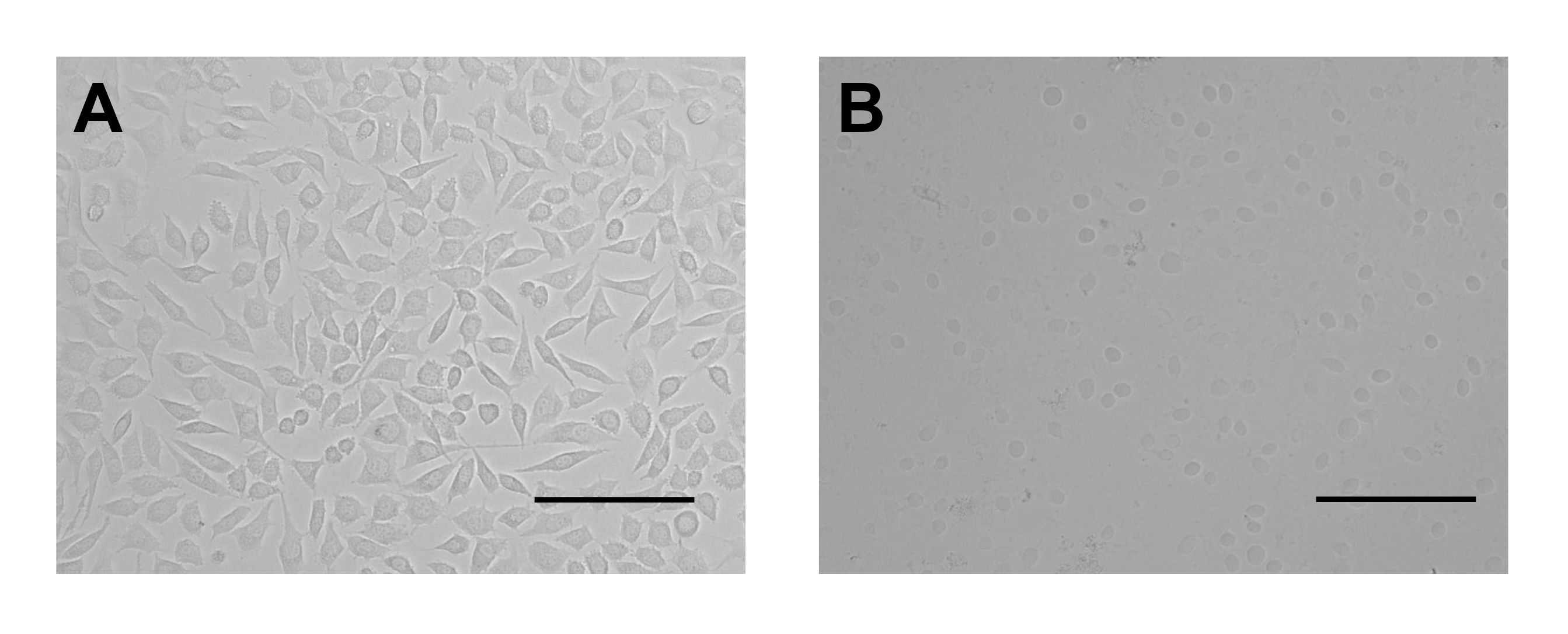

Supplement: Supplementary file 4 — Additional file 4: Figure S3. Cytotoxicity of L929 cells exposed to (A) complete medium (negative control) and (B) Triton-X-100 (positive control). Scale bar: 250 µm. [file 12906_2024_4381_MOESM4_ESM.jpg]
